# Deterioration of health-related quality of life: the hidden health burden of informal caregiving

**DOI:** 10.1007/s10198-025-01776-5

**Published:** 2025-04-17

**Authors:** Syed Afroz Keramat, Prianka Maria Sarker, Tracy Comans, Deborah Brooks, Nadeeka N. Dissanayaka

**Affiliations:** 1 Centre for Health Services Research, Faculty of Health, Medicine and Behavioural Sciences, The University of Queensland, Brisbane, Australia; 2 Margaret Chase Smith Policy Center, University of Maine, Orono, ME USA; 3 The University of Queensland Centre for Clinical Research (UQCCR), Faculty of Health, Medicine and Behavioural Sciences, The University of Queensland, Brisbane, Australia

**Keywords:** Australia, Health-related quality of life, Informal caregiving, MCS, PCS, SF-6D utility index

## Abstract

Informal caregiving is physically and mentally demanding and may lead to poor health and impaired well-being. We aim to check the effects of informal caregiving on health-related quality of life (HRQoL). We utilised longitudinal data from the most recent sixteen waves of the Household, Income and Labour Dynamics in Australia (HILDA) Survey. We measured HRQoL through the physical component summary (PCS), the mental component summary (MCS), the short-form six-dimension (SF-6D) utility index, and the eight dimensions of the SF-36 health survey. The primary exposure variable is informal caregiving. The variable was categorised into not a caregiver, lighter (< 5 hours [h]/week), moderate (5–19 h/week), and intensive (≥ 20 h/week) caregiving based on the hours of providing informal care per week. We have found that informal caregiving negatively affects HRQoL. We found that moderate (β = -0.0035, standard error [SE] = 0.0012) and intensive caregiving (β = -0.0074, SE = 0.0020) reduced SF-6D utility value. We also found that lighter (β = -0.29, SE = 0.07), moderate (β = -0.55, SE = 0.12), and intensive (β = -1.53, SE = 0.19) caregiving lowered MCS scores. Moreover, our results revealed that lighter, moderate and intensive caregiving decreases the scores of mental health-related dimensions (e.g., role emotional, social functioning, and mental health) of the SF-36 health survey. Our study findings have significant policy implications for improving the HRQoL and well-being of caregivers. To effectively support the health and well-being of informal carers, it is crucial to implement strategies that address their diverse needs, including supporting work-life balance, providing financial assistance and social security measures, ensuring access to community-based services, providing access to relevant information and training, and offering respite care options.

**Supplementary Information:**

The online version contains supplementary material available at 10.1007/s10198-025-01776-5.

## Introduction

Informal caregiving is increasingly becoming an essential part of the modern healthcare system as many countries are experiencing rapid increases in the ageing population (e.g., people aged ≥ 65 years) and a resulting higher prevalence of chronic diseases [1]. Informal caregiving is usually described as unpaid assistance or care provided by family members, friends, or relatives to older people or people with chronic health conditions, mental illness, or disabilities. According to the Survey of Disability, Ageing and Carers, around 2.65 million people in Australia were involved with informal caregiving in 2018 [2]. An estimated 3.5% of the Australian population served as primary carers, with 72% of them being female [3]. In the OECD countries, around 13% of people aged 50 years and over reported providing informal care at least once a week in 2019. For these countries, on average, 62% of those reporting daily caregiving were female [4]. The rate of the adult population providing informal care ranges approximately between 13.5 and 25.6% for European countries and 7-17% for the United States [5]. With increased demand for informal caregiving, the notion of caregivers’ health and well-being has also received substantial global research attention. Many empirical studies from the WHO European Region, Region of the Americas and the Western Pacific Region have documented a significant negative association between informal caregiving and caregivers’ physical and mental health. Several studies from the European region also found that informal caregiving increases perceived stress and depressive symptoms, lowers self-rated health and life satisfaction, impairs cognitive functioning, and decreases overall subjective well-being [6–13]. Additionally, studies conducted in the Western Pacific Region and the Region of the Americas have highlighted the observed adverse effects on caregivers’ psychological health [14–19]. Existing literature from the European and Western Pacific regions also provide evidence of noticeable gender differences, with women caregivers disproportionately experiencing greater stress, anxiety, caregiving burnout, poorer mental health, and lower life satisfaction [20–26]. Caregiving responsibilities often result in reduced time availability for caregivers’ own physical activity, leisure, or social activities, which extend some plausible reasonings for self-perceived poorer health among this population [8, 17, 22, 27]. Moreover, the extent, duration, and nature of caregiving and the relationship between caregiver and recipient also play a significant role in aggravating or extenuating adverse health consequences of informal caregiving [18, 28, 29].

Health-related quality of life (HRQoL) is a multidimensional comprehensive indicator that summarises an individual’s perceived physical and mental health status over time and examines the impact of health status on quality of life [30]. The concept encompasses assessments of self-rated physical and mental health conditions. Besides traditional health measures, HRQoL has increased its importance as a public health indicator for predicting health burdens and guiding health policy formulations. Several empirical studies have specifically explored the association between informal caregiving and HRQoL in the international and Australian context. For example, several studies conducted in several European countries used health state utility values to measure HRQoL. They found that caregivers had lower utility values compared to non-caregivers. Additionally, several studies have consistently shown that specific groups of caregivers, including those providing intensive care, female caregivers, and those caring for individuals with rare diseases, experience the most significant declines in HRQoL [6, 9, 31–35]. In Australia, several studies utilised data from the Household, Income and Labour Dynamics of Australia (HILDA) Survey to examine various components of HRQoL among caregivers. In general, these studies focused on physical functioning, general health, mental health or life satisfaction domains of the HRQoL and revealed a negative association between informal caregiving and HRQoL [15, 19, 36, 37]. These studies emphasise that specific groups of caregivers, including females, those providing high levels of care, and those caring for individuals with disabilities, are particularly vulnerable to experiencing significant declines in HRQoL.

The existing literature consistently demonstrates a strong association between informal caregiving and adverse health outcomes. However, prior research in Australia has been limited in scope, often focusing on specific components of HRQoL, such as mental health or physical function, rather than providing a comprehensive assessment of overall HRQoL. We extend the existing literature by providing a broader analysis of the effects of informal caregiving on HRQoL. To comprehensively assess the impact of informal caregiving on HRQoL, we utilised data from the most recent 16 waves of the HILDA Survey to examine the effects of informal caregiving on both the physical and mental component summary measures (PCS and MCS scores), SF-6D utility value, as well as each of the eight individual domains of the SF-36 health survey. To the best of our knowledge, this is the first longitudinal study in Australia that comprehensively examines the impact of informal caregiving on all eight dimensions of the SF-36 health survey. This comprehensive approach offers a more granular and complete picture of the relationships than previous studies that often focus on summary scores or a limited subset of domains. By dissecting the effects of caregiving across eight dimensions of the SF-36 health survey, we gain a deeper understanding of the specific facets of HRQoL that are severely affected. This detailed examination is crucial for developing targeted interventions and support systems that address the diverse needs of informal caregivers and mitigate the negative impacts on their well-being. Identifying the domains most vulnerable to the stresses of informal caregiving allows for the design of more effective and tailored support programs, ultimately leading to improved outcomes for informal caregivers. In addition to examining the eight dimensions of the SF-36, our study investigates the impact of informal caregiving on the SF-6D utility index, a crucial measure for economic evaluations of interventions aimed at improving the health and well-being of caregivers. Finally, by examining within-person differences in informal caregiving and HRQoL over time and exploring gender heterogeneity in the impact of informal caregiving, we aim to provide valuable insights for developing more targeted and effective policy interventions to support the diverse needs of informal caregivers.

## Methodology

### Data source

Our study utilised data from the HILDA Survey. The HILDA Survey is Australia’s largest household-based longitudinal study that collects valuable information about respondents’ demographic characteristics, household and family relationships, income, wealth, employment status, health, and educational outcomes. Since its inception in 2001, this nationally representative survey has followed the lives of more than 17,000 Australian adults each year. The survey is designed and governed by the Melbourne Institute of Applied Economic and Social Research. A multistage sampling procedure is used to collect data from individuals aged 15 years or older in each household using a self-completion questionnaire and in-person or telephone interviews by trained professional interviewers. Detailed information about the HILDA Survey design and data collection procedure is published elsewhere [38].

## Sample selection

We used data from the most recent sixteen waves (waves 6 [2006] through 21 [2021]) of the HILDA Survey. We have chosen the HILDA Survey for our study because it offers comprehensive information on informal caregiving, HRQoL, and detailed demographic and socio-economic characteristics of participants. The study period encompassed sixteen years, from 2006 to 2021, with wave 6 (2006) serving as the baseline and wave 21 (2021) representing the final wave of data. The study sample was restricted to Australian adults aged 15 years and over who completed the self-completion questionnaire. The primary reason is that the HILDA Survey gathered data each year through interviews with all household members aged 15 years and older. We did not exclude participants due to missing information on key variables across all sixteen waves of data. This approach helps maintain the national representativeness of the sample drawn from the nationally representative survey. A two-step approach was employed to handle missing data to minimise data loss. Firstly, for variables within the same individual across different survey waves, missing values were imputed using data from the preceding or subsequent wave when available. Subsequently, for any remaining missing values, simple imputation methods were applied, utilising the mean for continuous variables and the mode for categorical variables. The final analytic sample consists of 230,462 person-year observations from 27,957 unique individuals. A flowchart illustrating the analytical sample selection process, including the application of exclusion criteria, is provided in Appendix Figure A1. Additionally, Appendix Table A1 provides a detailed summary of the patterns and characteristics of missing data for all variables included in the analysis.

## Measures

### Dependent variables

HRQoL served as the primary outcome of interest in our study. The HILDA Survey collects information on HRQoL through RAND Corporation’s 36-item Short Form Survey (SF-36). The SF-36 is a widely used, easily comprehendible questionnaire that contains 36 questions to capture various domains of individuals’ physical and mental health conditions. A total of thirty-five items were utilised to construct eight distinct scales. An additional item specifically assessed health transition. The 35 items are specifically designed to determine eight different dimensions of health, including Physical Functioning (PF), Role Physical Functioning (RP), Role Emotional Functioning (RE), Social Functioning (SF), Mental Health (MH), Vitality (VT), Bodily Pain (BP), and General Health (GH), which are considered mutually exclusive. Responses on the eight health dimensions are scored on a scale of 0 to 100. Higher scores on each dimension indicate a higher level of HRQoL [39]. To provide a more comprehensive assessment of overall health, the eight individual health dimensions are further grouped into two summary measures: the Physical Component Summary (PCS) and the Mental Component Summary (MCS). The PCS and MCS measures are transformed from the eight dimensions using the appropriate scoring algorithm recommended [40], and scores on both scales are standardised using a linear z-score transformation with a mean of 50 and a standard deviation of 10. In our study, the scores for PCS and MCS ranged from 3.85 to 77.98 and − 3.19 to 76.88, respectively. As with the specific individual dimensions, higher scores on the PCS and MCS indicate improved HRQoL. Additionally, our study considers a measure of health utility index, SF-6D, as a major outcome of interest. Besides the SF-36 sub-scales, the HILDA Survey also contains this derived measure, which estimates each respondent’s preference for a particular health status. This approach facilitates measuring the utility of individuals from different health dimensions. The SF-6D utility index comprises six dimensions of the SF-36 health survey: PF, RP, RE, SF, VT, and BP. The value of the SF-6D utility index ranges between 0.29 and 1, where 0.29 indicates the worst possible health state, and 1 reflects the best possible health state [41].

## Exposure variable

Informal caregiving is typically defined as providing voluntary assistance to family members, friends, neighbours, or relatives who require support due to chronic illness or disability. The HILDA Survey identifies respondents as informal caregivers if they respond positively to whether they currently provide assistance or care to a family member with a long-term health condition, disability, or ageing. Additionally, the survey measures caregivers’ time allocation to caregiving responsibilities in a typical week, using follow-up questions. Based on the self-completion questionnaire responses related to caregiving commitments, we developed a four-level categorical exposure variable: not a caregiver (0 hours/week), lighter caregiving (< 5 hours/week), moderate caregiving (5–19 hours/week) and intensive caregiving (20 or more hours/week) in line with two prior studies on informal caregiving [36, 42]. We examined the transition probabilities between different levels of informal caregiving. Appendix Table A3 presents the transition rates for individuals moving from ‘not a caregiver’ to ‘intensive caregiving’ for each level of informal caregiving.

### Control variables

To account for potential confounding factors, we incorporated a comprehensive set of control variables, including socio-demographic characteristics, health-related factors, health-related behavioural characteristics, and a range of stressful life events following prior research [9, 15, 19, 33, 36, 37]. Socio-demographic controls included age, relationship status, highest education level completed, labour market status, household yearly disposable income, and region of residence. We included two health-related characteristics (weight and disability status) and three health-related behavioural characteristics (smoking, alcohol consumption, and physical activity). We also included three broad types of stressful life events in each regression model: work-related, family-related, and personal. Stressful life events related to work include retiring from the workforce, losing a job due to being fired or made redundant, and experiencing major worsening in financial situation. Family-related stressful life events considered in our study included serious illness or injury to a family member, the death of a spouse or child, and the death of a close relative/family member. Personal stressful life events included experiencing personal injury or illness, as well as experiencing physical violence. A detailed description of all the control variables is provided in Appendix Table A2.

## Estimation strategy

We constructed an unbalanced panel dataset containing 230,462 person-year observations derived from linking the de-identified records of 27,957 unique individuals who participated in at least one of the study’s waves (waves 6 through 21). The descriptive analysis summarised analytical sample characteristics. The descriptive statistics were reported as frequency (n) and percentage (%) for categorical variables and mean and standard deviation (SD) for the continuous variables.

We employed the fixed-effects regression models as our primary estimation strategy to identify the within-person differences in the association between informal caregiving and HRQoL. The fixed-effects models allow us to compare within-person changes in an individual’s HRQoL during different observation periods of varying caregiving responsibilities. The fixed-effects models inherently control for time-invariant unobserved characteristics that could otherwise potentially lead to omitted-variable biased estimates. The estimated regression model has the following functional form.

1 $$\begin{aligned}&HRQo{L_{it}} - \:\overline {HRQo{L_i}} = \beta {\:_1}(I{C_{it}} - \overline {I{C_i}} ) \\& + \beta {\:_2}\:\left( {{X_{it}} - \:\overline {{X_i}} } \right) + \left( {{\varepsilon _{it}} - \:\overline {{\varepsilon _i}} \:} \right) \\\end{aligned} $$

In Eq. (1), HRQoL represents the dependent variables of interest; IC denotes the exposure variable (informal caregiving); and X refers to the vector of control variables. $\:{\beta\:}_{1}$ and $\:{\beta\:}_{2}$ are the vectors of model coefficients to be estimated, and $\:{ϵ}_{it}$ stands for the error term. In Eq. (1), the subscripts i and t refer to individual and time, respectively.

We fitted three sets of regression models for our key outcome variables: PCS, MCS, and SF-6D. Further, we estimated separate regression models for each of the eight dimensions of the SF-36 health survey: PF, RP, RE, SF, MH, VT, BP, and GH. We controlled socio-demographic characteristics, health-related factors, behaviours, and stressful life events in each regression model.

Various factors can be pivotal in shaping the relationship between informal caregiving and HRQoL. In particular, health-related characteristics (e.g., disability) and social determinants of health (e.g., labour force status) may substantially impact caregiving and an individual’s HRQoL. A more in-depth analysis of the interaction between these factors (disability and labour force status) with informal caregiving and their effects on HRQoL may provide valuable insights for targeted interventions and policy development. To investigate the interplay between informal caregiving, disability status, and labour force status on HRQoL, we conducted group comparisons of their interaction effects on several key HRQoL indicators: PCS, MCS, the SF-6D utility value, and the eight dimensions of the SF-36 health survey.

To assess the robustness of our findings, sensitivity analyses were conducted by re-running all regression models using fixed-effects models, excluding data from the COVID-19 period (2020 [wave 20] and 2021 [wave 21]). To explore potential heterogeneity in the impact of informal caregiving on HRQoL across genders, we conducted subgroup analyses by re-estimating our primary models separately for male and female respondents.

We set the p-value at 5% or lower for statistical significance for each explanatory variable. All statistical analyses were conducted using Stata Statistical Software (Release 17). College Station, TX: StataCorp LLC.

## Results

### Descriptive statistics

Table 1 presents the distribution of participants across various socio-demographic characteristics, health-related factors, health-related behaviours, and exposure to stressful life events. The results indicated that most participants were middle-aged adults (39.68%), female (53.29%), lived with a partner (59.42%), had an education level of Year 12 or below (43.55%), were employed (63.33%), identified as non-Indigenous (97.41%), and resided in major cities (65.95%). The table further reveals that approximately 24.11% of participants were obese, 28.65% had a disability, 17.90% were current smokers, 80.76% consumed alcohol, and 66.44% did not perform recommended levels of physical activity. The results also showed that many participants experienced significant life stressors. This included 3.21% who were made redundant, 14.62% who reported a serious illness or injury within their family, 11.83% who experienced the loss of a close relative or family member, and 8.93% who suffered a personal injury (pooled data).

**Table 1:** Descriptive statistics: analysis sample from HILDA (waves 6, wave 21, and pooled data)

Parameters	Baseline (Wave 6)		Final timepoint (Wave 21)		Pooled data (Wave 6 through 21)		
	*n*	%	*n*	%	*n*	%	
**Socio-demographic characteristics**							
**Age**							
15–24 years (Youth)	2,115	18.10	2,165	14.13	38,732	16.81	
25–39 years (Young adult)	2,869	24.55	4,221	27.55	57,898	25.12	
40–64 years (Middle-aged adult)	4,829	41.32	5,680	37.07	91,451	39.68	
≥ 65 years (Older adult)	1,875	16.04	3,255	21.25	42,381	18.39	
**Gender**							
Male	5,449	46.62	7,072	46.16	107,638	46.71	
Female	6,239	53.38	8,249	53.84	122,824	53.29	
**Relationship status**							
Partnered	6,888	58.93	9,158	59.77	136,952	59.42	
Unpartnered	4,800	41.07	6,163	40.23	93,510	40.58	
**Highest education level completed**							
Year 12 and below	6,003	51.36	5,716	37.31	100,361	43.55	
Professional qualifications	3,229	27.63	5,032	32.84	71,818	31.16	
University qualifications	2,456	21.01	4,573	29.85	58,283	25.29	
**Labour market status**							
Employed	7,547	64.57	9,699	63.31	145,962	63.33	
Unemployed/NLF	4,141	35.43	5,622	36.69	84,500	36.67	
**Household yearly disposable income**							
Quintile 1 (Poorest)	2,338	20	3,065	20.01	46,095	20	
Quintile 2 (Poorer)	2,338	20	3,065	20.01	46,091	20	
Quintile 3 (Middle)	2,337	19.99	3,063	19.99	46,094	20	
Quintile 4 (Richer)	2,341	20.03	3,065	20.01	46,091	20	
Quintile 5 (Richest)	2,334	19.97	3,063	19.99	46,091	20	
**Indigenous status**							
Not of Indigenous origin	11,455	98.01	14,846	96.9	224,484	97.41	
Indigenous origin	233	1.99	475	3.10	5,978	2.59	
**Region of residence**							
Major city	7,504	64.2	10,035	65.50	151,992	65.95	
Regional city and remote area	4,184	35.8	5,286	34.50	78,470	34.05	
**Health-related characteristics**							
**Weight category (BMI)**							
Underweight	359	3.07	327	2.13	5,873	2.55	
Healthy weight	4,905	41.97	5,254	34.29	88,215	38.28	
Overweight	4,051	34.66	5,456	35.61	80,802	35.06	
Obese	2,373	20.30	4,284	27.96	55,572	24.11	
**Disability**							
No	8,581	73.42	10,555	68.89	164,427	71.35	
Yes	3,107	26.58	4,766	31.11	66,035	28.65	
**Health-related behavioural characteristics**							
**Smoking status**							
Non-smoker	9,205	78.76	13,050	85.18	189,200	82.10	
Current smoker	2,483	21.24	2,271	14.82	41,262	17.90	
**Alcohol consumption**							
Non-drinker	2,002	17.13	3,093	20.19	44,341	19.24	
Current drinker	9,686	82.87	12,228	79.81	186,121	80.76	
**Physical activity**							
Less than the recommended level	7,694	65.83	10,042	65.54	153,130	66.44	
Recommended level	3,994	34.17	5,279	34.46	77,332	33.56	
**Work-related stressful life events**							
**Retiring from the workforce**							
No	11,394	97.48	14,952	97.59	224,579	97.45	
Yes	294	2.52	369	2.41	5,883	2.55	
**Getting fired or made redundant**							
No	11,348	97.09	14,872	97.07	223,061	96.79	
Yes	340	2.91	449	2.93	7,401	3.21	
**Major worsening in financial situation**							
No	11,380	97.36	15,010	97.97	223,932	97.17	
Yes	308	2.64	311	2.03	6,530	2.83	
**Family-related stressful life events**							
**Serious injury/illness to family member**							
No	9,786	83.73	13,366	87.24	196,773	85.38	
Yes	1,902	16.27	1,955	12.76	33,689	14.62	
**Death of spouse or child**							
No	11,582	99.09	15,205	99.24	228,484	99.14	
Yes	106	0.91	116	0.76	1,978	0.86	
**Death of a close relative/family member**							
No	10,425	89.19	13,561	88.51	203,197	88.17	
Yes	1,263	10.81	1,760	11.49	27,265	11.83	
**Personal stressful life events**							
**Personal injury or illness to self**							
No	10,658	91.19	14,085	91.93	209,873	91.07	
Yes	1,030	8.81	1,236	8.07	20,589	8.93	
**Experience of physical violence**							
No	11,486	98.27	15,122	98.70	227,082	98.53	
Yes	202	1.73	199	1.30	3,380	1.47	

*Note: (1) In the pooled data*,* a total of 230*,*462 yearly observations were considered from 27*,*957 unique persons. (2) The study used a ‘modified OECD’ equivalence scale to measure equivalised household annual disposable income and then categorised it into quintiles. (3) Values are rounded off to two decimal places. (4) Abbreviation: NLF = Not in the labour force.*

Table 2 presents the summary statistics of the participants’ health scores measured through the SF-36 health survey and informal caregiving status at baseline, the final wave, and all waves pooled. The mean scores for the PCS, MCS, and SF-6D utility values were 49.17, 48.05, and 0.75, respectively (pooled data). The results indicated that the mean scores on four individual dimensions of the SF-36 health survey exceeded 75 points on a scale of 100: physical functioning (PF) at 83.11, role physical (RP) at 77.95, role emotional (RE) at 81.24, and social functioning (SF) at 81.51. Table 2 also shows that 12.80% of participants engaged in some form of informal caregiving. Specifically, 7.51% provided lighter caregiving, 3.46% offered moderate caregiving, and 1.83% involved intensive caregiving (pooled data).

**Table 2:** Summary statistics: subjective health scores and informal caregiving

Parameters	Baseline (Wave 6)		Final timepoint (Wave 21)		Pooled data (Wave 6 through 21)	
	*n*	Mean (SD)	*n*	Mean (SD)	*n*	Mean (SD)
**SF-36 domain scores**						
Physical functioning	11,688	83.29 (23.44)	15,321	15.321 (83.48)	230,462	83.11 (23.71)
Role physical	11,688	79.07 (35.97)	15,321	76.46 (37.18)	230,462	77.95 (36.70)
Role emotional	11,688	83.22 (32.51)	15,321	75.60 (37.77)	230,462	81.24 (34.34)
Social functioning	11,688	82.54 (23.53)	15,321	79.02 (25.31)	230,462	81.51 (24.08)
Mental health	11,688	74.28 (17.12)	15,321	70.17 (18.42)	230,462	73.25 (17.63)
Vitality	11,688	60.42 (19.65)	15,321	55.80 (20.67)	230,462	58.87 (20.11)
Bodily pain	11,688	73.56 (24.24)	15,321	71.28 (23.44)	230,462	72.27 (24.09)
General health	11,688	68.74 (21.21)	15,321	65.84 (20.45)	230,462	67.35 (20.95)
**SF-36 component summary scores and utility values**						
PCS	11,688	49.37 (10.44)	15,321	49.59 (10.60)	230,462	49.17 (10.56)
MCS	11,688	48.81 (10.27)	15,321	45.85 (11.65)	230,462	48.05 (10.81)
SF-6D	11,688	0.76 (0.12)	15,321	0.74 (0.12)	230,462	0.75 (0.12)
**Main exposure variable**						
**Informal caregiving**						
Not a caregiver	10,096	86.38	12,941	84.47	200,960	87.20
Lighter caregiving	1,017	8.70	1,596	10.42	17,318	7.51
Moderate caregiving	384	3.29	506	3.30	7,968	3.46
Intensive caregiving	191	1.63	278	1.81	4,216	1.83

*Notes: (1) In the pooled data*,* a total of 230*,*462 yearly observations were considered from 27*,*957 unique persons. (2) Abbreviations: PCS = Physical component summary*,* MCS = Mental component summary*,* and SD = Standard deviation.*

**Fig. 1:**
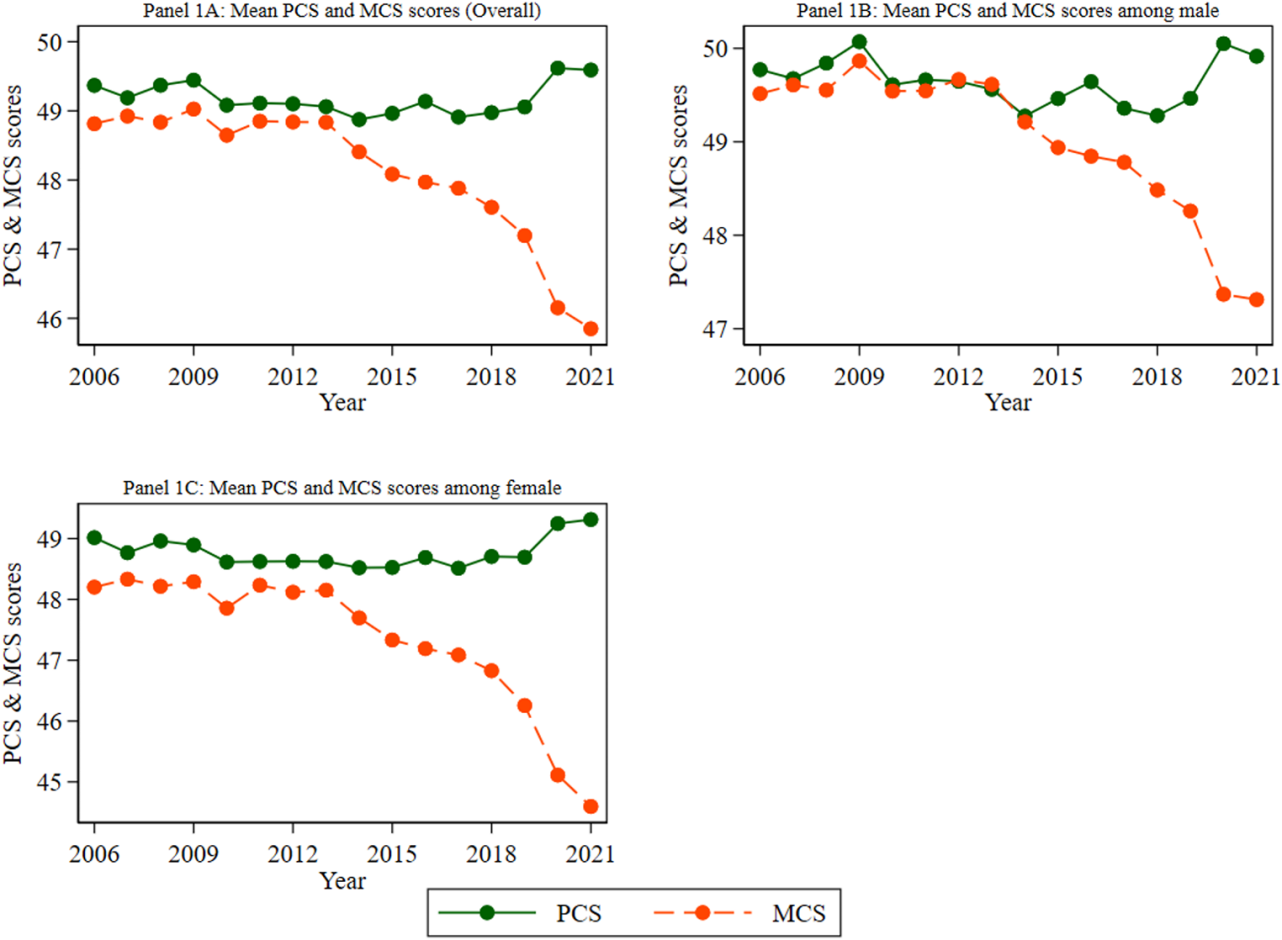
Trends in the overall mean PCS and MCS scores, as well as by gender. Notes: 1. Abbreviations: PCS = Physical component summary and MCS = Mental component summary

Figure 1 illustrates the trend in mean scores for the PCS, MCS, and the SF-6D utility index over the study period spanning from 2006 to 2021 (Panel 1 A). Figure 1 (Panels 1B and 1 C) also shows the trends in PCS, MCS, and SF-6D utility values specifically for male and female participants throughout the study period (2006–2021). We observed that the mean PCS scores consistently exceeded the mean MCS scores each year. Specifically, from wave 14, the mean MCS scores have been continuously declining. However, the mean PCS scores followed a relatively stable upward trend. A notable divergence emerged from wave 14 onwards. While the mean MCS score exhibited a consistent decline, the mean PCS score demonstrated a relatively stable and even slightly upward trajectory. The gap between the two summary scores was more pronounced in the final two waves of the survey. For instance, at the final time point (wave 21), the mean PCS and MCS scores were 49.59 and 45.85, respectively (Panel 1A). While both men and women exhibited similar trends in PCS and MCS scores over the study period, a slight but consistent difference was observed. Men generally reported slightly higher scores in both domains. The decline in average MCS scores, particularly noticeable in the last two years of the study (2020 and 2021), could be attributed to the effects of the COVID-19 pandemic. Several research studies from Australia, Europe, Japan and the United States have found that COVID-19 increased the mental health burden of informal caregivers as they experienced heightened levels of uncertainty and anxiety, increased caregiving responsibilities and a lack of social support [43–48].

**Fig. 2:**
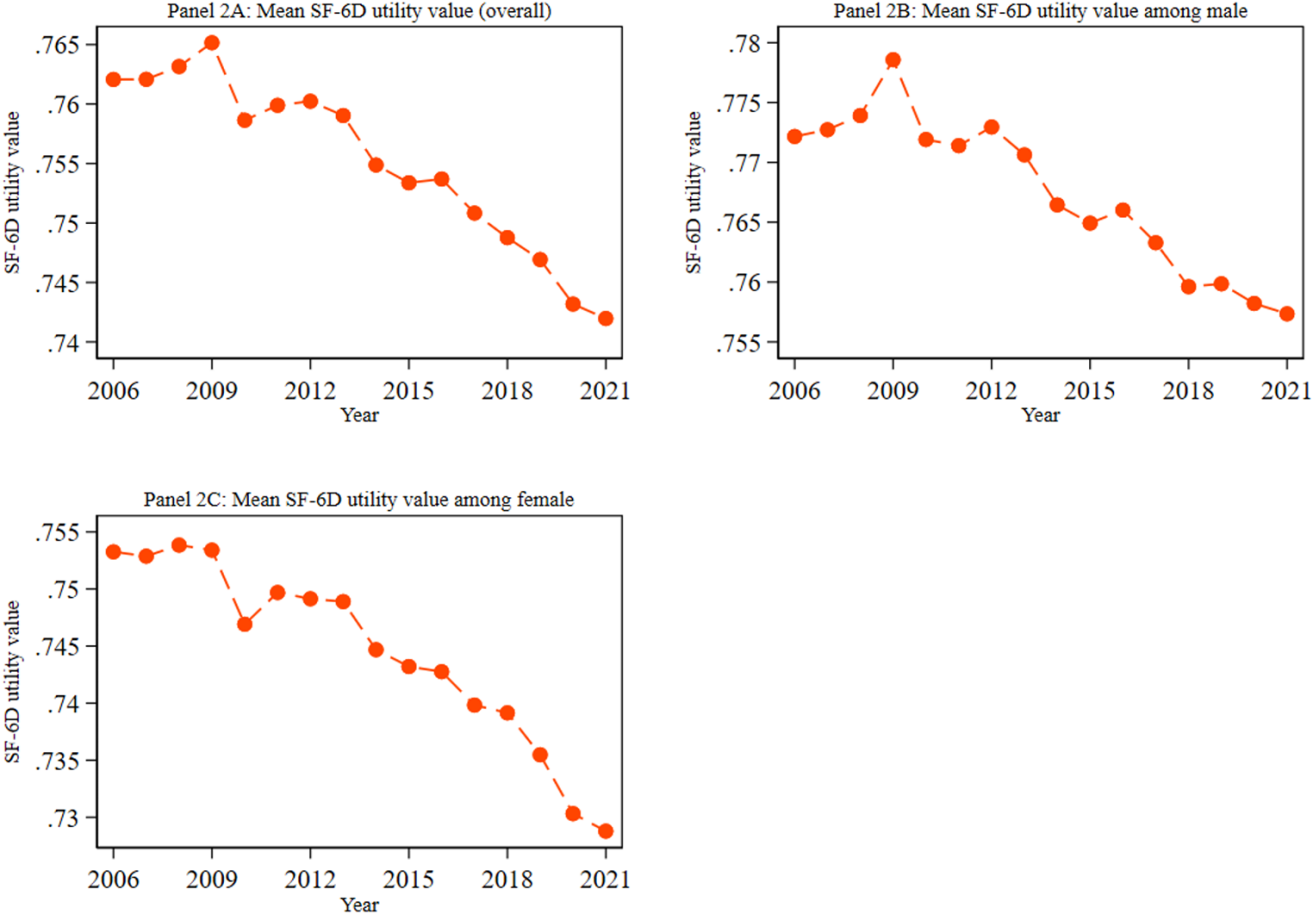
Trend in the overall mean SF-6D utility value, as well as by gender. Notes: 1. Abbreviations: SF-6D = Short-Form Six-Dimension

Figure 2 illustrates how the average SF-6D utility value changes over time and displays the differences between males and females in these trends. Figure 2 reveals a gradual decline in the average SF-6D utility value, with values observed between 0.76 in 2006 and 0.74 in 2021 (Panel 2 A). The figure demonstrates a consistent downward trend in the average SF-6D utility value for men and women across the study period. While both genders exhibited this decline, men consistently maintained a slightly higher average SF-6D utility value than women in each measurement wave (Panels 2B and 2 C). For instance, the average SF-6D utility values were 0.757 for men and 0.729 for women in 2021. Earlier, we observed a decline in MCS score (Fig. 1). Therefore, the declining trend in mean SF-6D utility value may be due to the decline in mental health components of the SF-36. The observed decrease in the average SF-6D utility value may be linked to the decline in mental health. The deterioration of mental health dimensions, as measured by the SF-36 health survey, could contribute to the lower overall quality of life scores represented by the SF-6D utility values.

**Fig. 3:**
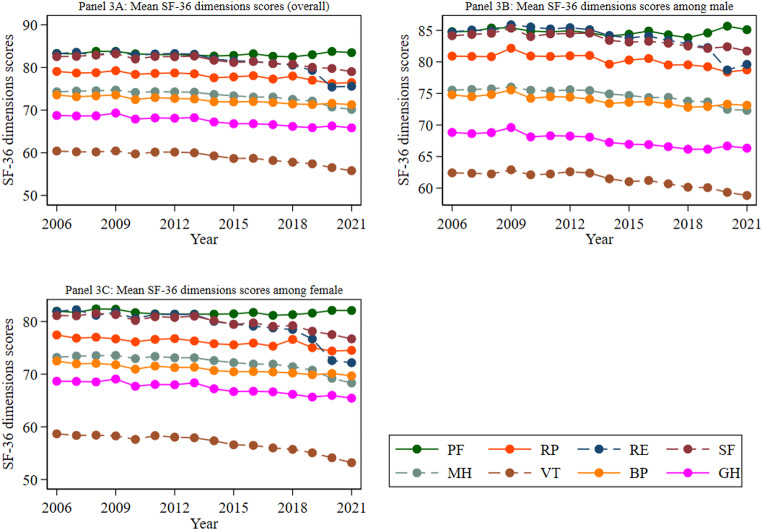
Trend in the mean SF-36 dimensions scores overall and by gender. *Notes: 1. Abbreviations: physical functioning (PF)*,* role physical functioning (RP)*,* role emotional functioning (RE)*,* social functioning (SF)*,* mental health (MH)*,* vitality (VT)*,* bodily pain (BP) and general health (GH)*

Figure 3 depicts the trends in the mean scores across the eight distinct domains (PF, RP, RE, SF, MH, VT, BP, GH) of the SF-36 health survey, with separate trends presented for men and women. The domains of vitality (less than 60) and general health (less than 70) consistently exhibited lower mean scores compared to the remaining SF-36 dimensions throughout the study period. This trend also holds for both men and women.

**Fig. 4:**
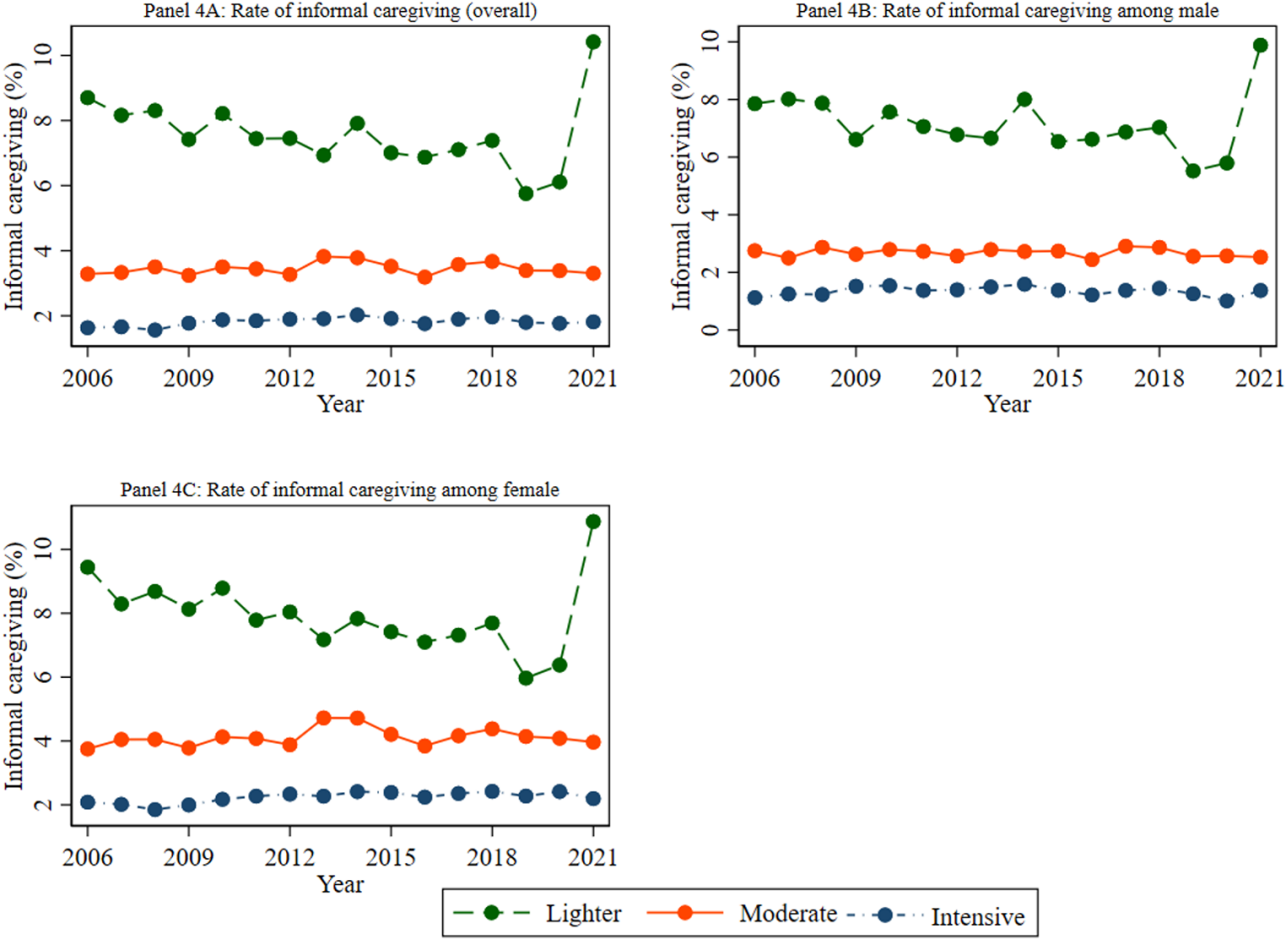
Estimated rates of informal caregiving overall and by gender

Figure 4 depicts the pattern of informal caregiving among study participants over the past sixteen waves of the HILDA Survey, highlighting gender-specific trends. The figure revealed that respondents engaged in informal caregiving varied from 13.62% in 2006 to 15.53% in 2021 (Panel 4 A). Among individuals providing informal care, the rate of lighter caregiving was the highest, followed by moderate and intensive caregiving. This pattern was consistent for both male and female caregivers. For instance, the figure (Panel 4 A) showed that a higher percentage of respondents provided lighter care (10.42%) compared to moderate care (3.30%) and intensive care (1.81%) in 2021. Figure 4 (Panels 4B and 4 C) also demonstrates that women consistently provided more informal care than men throughout the study period. For example, a higher percentage of women (2.19%) provided intensive care than men (1.37%) in 2021.

**Fig. 5:**
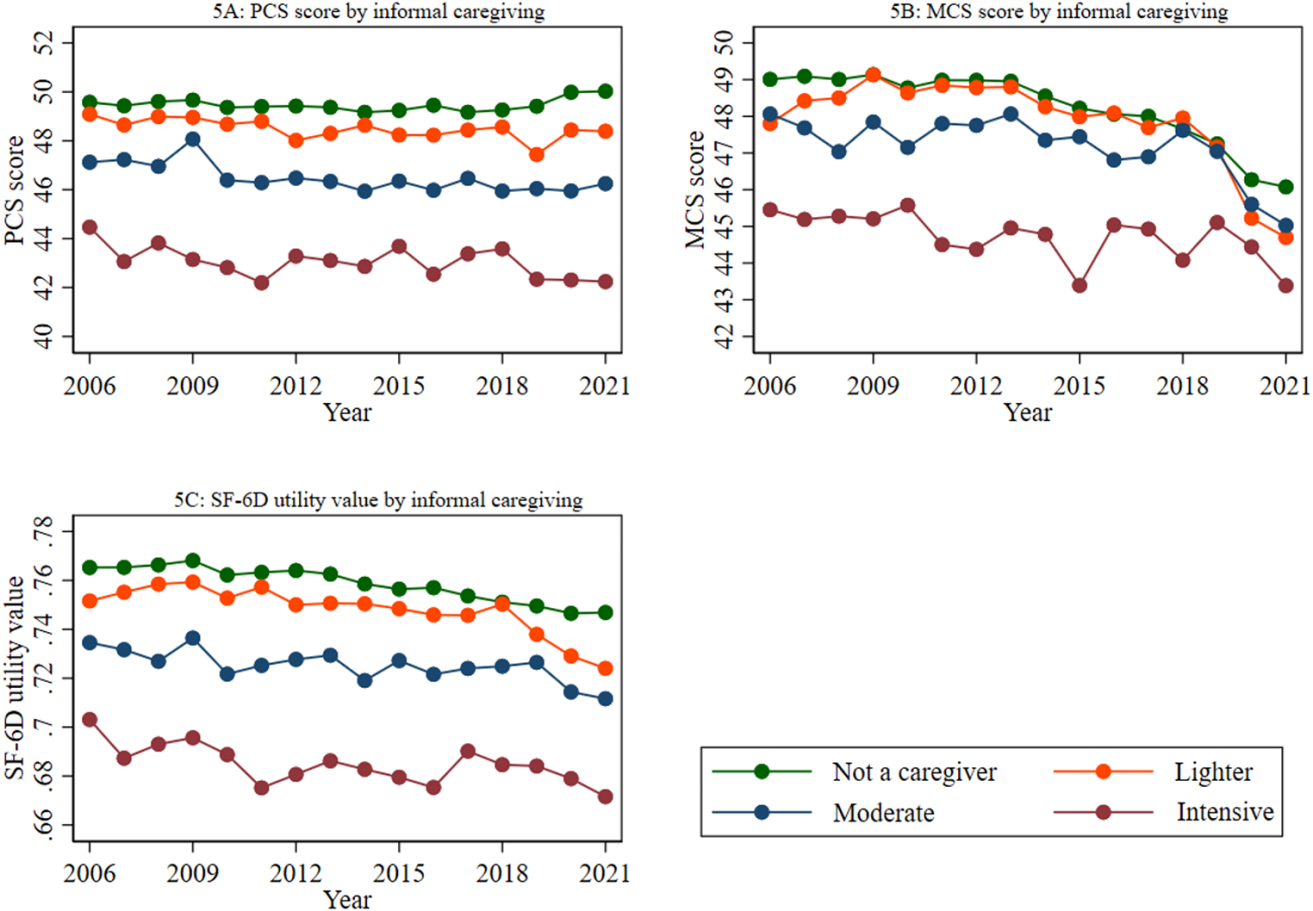
Trend in the mean PCS, MCS, and SF-6D utility values by informal caregiving. *Notes: 1. Abbreviations: PCS*,* Physical component summary; MCS*,* Mental component summary*,* and SF-6D*,* Short-Form Six-Dimension.*

Figure 5 illustrates how HRQoL measures (PCS, MCS, and SF-6D utility index) change over time for individuals providing different levels of informal care. The figure demonstrated an inverse relationship between informal caregiving intensity and HRQoL. In 2021, mean PCS scores exhibited a clear downward trend, with non-caregivers (50.03) showing the highest scores, followed by a gradual decline among lighter caregivers (48.39), moderate caregivers (46.25), and a more pronounced decline among intensive caregivers (42.24) (Panel 5 A). In 2021, a similar pattern was observed for mean MCS scores, with non-caregivers (46.08) exhibiting the highest scores, followed by lighter caregivers (44.70), moderate caregivers (45.03), and intensive caregivers (43.38) (Panel 5B). The figure also showed that the mean SF-6D utility value is lowest among individuals offering intensive caregiving (0.67), followed by those offering moderate (0.71), lighter (0.72), and no caregiving responsibilities (0.75) in 2021 (Panel 5 C).

**Fig. 6:**
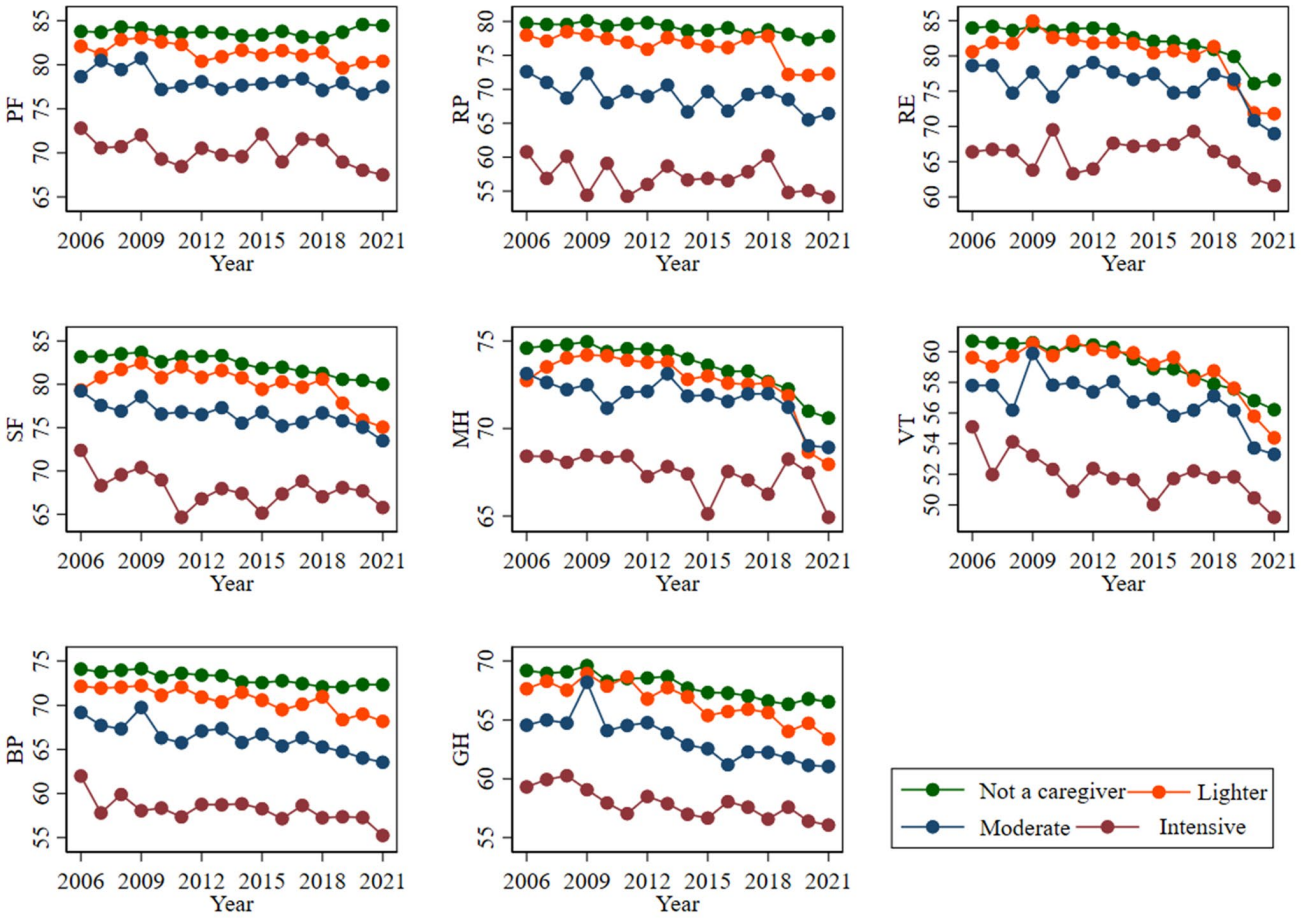
Trend in the mean SF-36 dimensions scores by informal caregiving.* Notes: 1. Abbreviations: PF = physical functioning*,* RP = role physical*,* RE = role emotional*,* SF = social functioning*,* MH = mental health*,* VT = vitality*,* BP = bodily pain*,* and GH = general health*

Figure 6 presents the trends in mean scores for the eight SF-36 dimensions (PF, RP, RE, SF, MH, VT, BP, GH) across different levels of informal caregiving. The figure showed that participants with no caregiving or lighter caregiving responsibilities typically exhibited higher mean scores on most SF-36 dimensions than those with moderate or intensive caregiving responsibilities. More specifically, individuals providing intensive care demonstrated the lowest mean scores across all eight SF-36 subscales. For example, in the final wave, mean GH scores decreased progressively from 66.54 for non-caregivers to 63.40 for lighter caregivers, 61.05 for moderate caregivers, and 56.07 for intensive caregivers.

### Regression estimates

Table 3 presents the estimated effects of informal caregiving on the HRQoL measures (PCS, MCS, and SF-6D) based on the fixed-effects regression modelling. After controlling for the socio-demographic, health-related characteristics, and stressful life events, we found that informal caregiving negatively affected the MCS score and SF-6D health state utility value. The regression results suggest that informal caregiving significantly reduced the MCS score and SF-6D utility value. Model 2 revealed that lighter, moderate and intensive caregiving decreased MCS scores by 0.29 (β = -0.29 [0.07]), 0.55 (β = -0.55 [0.12]) and 1.53 (β = -1.53 [0.19]) unit points, respectively. Model 3 also demonstrated that moderate and intensive caregiving significantly decreased SF-6D utility values by 0.0035 (β = -0.0035 [0.0012]) and 0.0074 (β = -0.0074, [0.0020]) unit points, respectively.

**Table 3:** Estimated effects of informal caregiving on the PCS, MCS, and SF-6D utility value; fixed-effects panel regression models

Models	(Model 1)	(Model 2)	(Model 3)
Variables	PCS	MCS	SF-6D utility index
Informal caregiving			
Not a caregiver (ref)			
Lighter caregiving	0.15*	-0.29***	0.0001
	[0.06]	[0.07]	[0.0008]
Moderate caregiving	0.20*	-0.55***	-0.0035**
	[0.10]	[0.12]	[0.0012]
Intensive caregiving	0.60***	-1.53***	-0.0074***
	[0.16]	[0.19]	[0.0020]
**Socio-demographic characteristics**			
**Age**			
15–24 years (ref)			
25–39 years	1.52***	-1.41***	0.0005
	[0.09]	[0.12]	[0.0013]
40–64 years	2.12***	-1.43***	0.0021
	[0.14]	[0.18]	[0.0018]
≥ 65 years	1.98***	-0.06	0.0119***
	[0.18]	[0.22]	[0.0023]
**Relationship status**			
Partnered (ref)			
Unpartnered	0.17*	-0.65***	-0.0048***
	[0.07]	[0.10]	[0.0010]
**Highest education level completed**			
Year 12 and below (ref)			
Professional qualifications	0.56***	-0.40*	-0.0006
	[0.12]	[0.16]	[0.0017]
University qualifications	0.64***	-0.44*	-0.0024
	[0.12]	[0.19]	[0.0018]
**Labour market status**			
Employed (ref)			
Unemployed/NLF	-0.74***	-0.41***	-0.0101***
	[0.06]	[0.07]	[0.0008]
**Household yearly disposable income**			
Quintile 1 (Poorest)	-0.11	-0.51***	-0.0046***
	[0.07]	[0.09]	[0.0010]
Quintile 2 (Poorer)	-0.23***	-0.42***	-0.0040***
	[0.06]	[0.08]	[0.0008]
Quintile 3 (Middle)	-0.19***	-0.23***	-0.0023**
	[0.06]	[0.07]	[0.0007]
Quintile 4 (Richer)	-0.14**	-0.12*	-0.0017**
	[0.05]	[0.06]	[0.0006]
Quintile 5 (Richest) (ref)			
**Region of residence**			
Major city (ref)			
Regional city and remote area	-0.03	0.28*	0.0016
	[0.10]	[0.12]	[0.0013]
**Health-related characteristics**			
**Weight category (BMI)**			
Underweight	-0.23	-0.56**	-0.0048**
	[0.13]	[0.18]	[0.0018]
Healthy weight (ref)			
Overweight	-0.28***	0.17*	-0.0015*
	[0.05]	[0.07]	[0.0007]
Obese	-1.10***	-0.08	-0.0095***
	[0.09]	[0.10]	[0.0011]
**Disability**			
No (ref)			
Yes	-3.20***	-1.48***	-0.0325***
	[0.06]	[0.07]	[0.0007]
**Health-related behavioural characteristics**			
**Smoking status**			
Non-smoker (ref)			
Current smoker	0.24**	-0.91***	-0.0063***
	[0.08]	[0.10]	[0.0011]
**Alcohol consumption**			
Non-drinker (ref)			
Current drinker	1.18***	-0.31***	0.0049***
	[0.07]	[0.08]	[0.0009]
**Physical Activity**			
Less than the recommended level (ref)			
Recommended level	1.03***	1.51***	0.0188***
	[0.04]	[0.05]	[0.0005]
**Work-related stressful life events**			
**Retiring from the workforce**			
No (ref)			
Yes	-0.40***	-0.05	-0.0017
	[0.10]	[0.11]	[0.0012]
**Getting fired or made redundant**			
No (ref)			
Yes	0.53***	-0.45***	-0.0007
	[0.09]	[0.11]	[0.0012]
**Major worsening in financial situation**			
No (ref)			
Yes	0.35**	-3.48***	-0.0234***
	[0.11]	[0.14]	[0.0013]
**Family-related stressful life events**			
**Serious injury/illness to family member**			
No (ref)			
Yes	0.14**	-0.72***	-0.0052***
	[0.04]	[0.05]	[0.0005]
**Death of spouse or child**			
No (ref)			
Yes	1.00***	-3.10***	-0.0205***
	[0.18]	[0.24]	[0.0023]
**Death of a close relative/family member**			
No (ref)			
Yes	0.07	-0.54***	-0.0045***
	[0.04]	[0.05]	[0.0006]
**Personal stressful life events**			
**Personal injury or illness to self**			
No (ref)			
Yes	-4.39***	-2.10***	-0.0491***
	[0.07]	[0.08]	[0.0008]
**Experience of physical violence**			
No (ref)			
Yes	0.42**	-2.49***	-0.0150***
	[0.15]	[0.21]	[0.0019]
Observations	230,462	230,462	230,462
Number of individuals	27,957	27,957	27,957
Individual FE	Yes	Yes	Yes
Time FE	Yes	Yes	Yes

Notes: (1) Robust standard errors in brackets. (2) ***, **, and * denote significance at the p < 0.01, *p < 0.05*,* and p < 0.1 levels*,* respectively. (3) Abbreviations: ref*,* reference category; PCS*,* Physical Component Summary; MCS*,* Mental Component Summary; SF-6D*,* Short-Form Six-Dimension health index.*

Table 4 summarises the observed effects of informal caregiving on the eight specific domains of the SF-36 health assessment tool (PF, RP, RE, SF, MH, VT, BP, and GH). The results suggest that informal caregiving exerted a more pronounced effect on the mental health domains (RE, SF, MH, and VT) of the SF-36 health survey. For example, intensive caregiving significantly reduced scores on RE (-3.84 [0.73]), SF (-2.25 [0.46]), MH (-1.92 [0.30]), and VT (-1.43 [0.32]) compared to those not providing any forms of informal caregiving.

**Table 4:** Estimated effects of informal caregiving on the dimensions of the SF-36 scores; fixed-effects panel regression models

Models	(Model 1)	(Model 2)	(Model 3)	(Model 4)	(Model 5)	(Model 6)	(Model 7)	(Model 8)
Variables	PF	RP	RE	SF	MH	VT	BP	GH
**Informal caregiving**								
Not a caregiver (ref)								
Lighter caregiving	0.16	0.18	-0.67*	-0.46**	-0.47***	-0.02	-0.06	-0.02
	[0.15]	[0.27]	[0.28]	[0.18]	[0.12]	[0.13]	[0.16]	[0.12]
Moderate caregiving	0.32	-0.30	-1.43***	-1.06***	-0.75***	-0.19	-0.10	-0.12
	[0.22]	[0.42]	[0.43]	[0.27]	[0.18]	[0.20]	[0.24]	[0.17]
Intensive caregiving	0.92*	-0.42	-3.84***	-2.25***	-1.92***	-1.43***	-0.14	-0.35
	[0.38]	[0.68]	[0.73]	[0.46]	[0.30]	[0.32]	[0.38]	[0.29]
**Socio-demographic characteristics**								
**Age**								
15–24 years (ref)								
25–39 years	3.26***	1.69***	-1.76***	-0.47	-1.86***	-1.57***	0.82***	1.60***
	[0.23]	[0.34]	[0.40]	[0.26]	[0.20]	[0.21]	[0.24]	[0.20]
40–64 years	4.91***	4.10***	-0.36	0.19	-2.25***	-0.73*	0.79*	1.83***
	[0.32]	[0.52]	[0.57]	[0.37]	[0.28]	[0.30]	[0.35]	[0.28]
≥ 65 years	5.11***	4.79***	2.78***	1.96***	-0.17	1.27**	1.74***	3.01***
	[0.42]	[0.73]	[0.74]	[0.48]	[0.34]	[0.38]	[0.45]	[0.35]
**Relationship status**								
Partnered (ref)								
Unpartnered	-0.91***	-0.33	-2.64***	-1.97***	-1.04***	0.75***	0.67***	0.00
	[0.17]	[0.27]	[0.32]	[0.20]	[0.15]	[0.16]	[0.18]	[0.14]
**Highest education level completed**								
Year 12 and below (ref)								
Professional qualifications	1.47***	0.62	-0.46	0.09	-0.50	-0.38	0.15	0.71**
	[0.30]	[0.42]	[0.50]	[0.32]	[0.25]	[0.27]	[0.31]	[0.26]
University qualifications	1.07***	1.09*	-1.04	0.22	-0.57*	-0.36	0.33	1.17***
	[0.27]	[0.45]	[0.59]	[0.35]	[0.29]	[0.32]	[0.33]	[0.29]
**Labour market status**								
Employed (ref)								
Unemployed/NLF	-1.80***	-4.00***	-3.64***	-2.21***	-0.54***	0.25	-1.02***	-0.56***
	[0.14]	[0.25]	[0.25]	[0.16]	[0.12]	[0.13]	[0.15]	[0.11]
**Household yearly disposable income**								
Quintile 1 (Poorest)	-0.59***	-0.91**	-1.49***	-0.77***	-0.80***	-0.92***	-0.36	-0.55***
	[0.17]	[0.31]	[0.32]	[0.20]	[0.14]	[0.16]	[0.19]	[0.14]
Quintile 2 (Poorer)	-0.84***	-1.05***	-1.10***	-0.85***	-0.64***	-1.06***	-0.40*	-0.59***
	[0.15]	[0.26]	[0.27]	[0.17]	[0.12]	[0.14]	[0.16]	[0.12]
Quintile 3 (Middle)	-0.49***	-0.72**	-0.37	-0.47**	-0.37***	-0.81***	-0.34*	-0.47***
	[0.12]	[0.23]	[0.23]	[0.15]	[0.11]	[0.12]	[0.14]	[0.11]
Quintile 4 (Richer)	-0.38***	-0.46*	-0.34	-0.32*	-0.21*	-0.40***	-0.32**	-0.24**
	[0.11]	[0.20]	[0.20]	[0.13]	[0.09]	[0.11]	[0.12]	[0.09]
Quintile 5 (Richest) (ref)								
**Region of residence**								
Major city (ref)								
Regional city and remote area	0.21	0.49	0.72	0.88***	0.36	0.12	-0.31	0.21
	[0.23]	[0.38]	[0.41]	[0.26]	[0.20]	[0.21]	[0.24]	[0.20]
**Health-related characteristics**								
**Weight category (BMI)**								
Underweight	-0.47	-1.22*	-1.57**	-1.44***	-0.86**	-0.47	-0.74*	-1.22***
	[0.33]	[0.54]	[0.59]	[0.38]	[0.28]	[0.30]	[0.34]	[0.27]
Healthy weight (ref)								
Overweight	-0.41**	-0.10	0.79***	0.55***	0.17	-0.64***	-0.43**	-0.67***
	[0.13]	[0.22]	[0.23]	[0.15]	[0.11]	[0.12]	[0.14]	[0.11]
Obese	-1.85***	-1.81***	0.17	-0.15	-0.19	-2.39***	-2.11***	-2.80***
	[0.21]	[0.34]	[0.36]	[0.23]	[0.16]	[0.17]	[0.21]	[0.17]
**Disability**								
No (ref)								
Yes	-5.77***	-11.56***	-6.47***	-5.60***	-2.64***	-4.22***	-6.94***	-5.70***
	[0.13]	[0.26]	[0.23]	[0.16]	[0.10]	[0.11]	[0.14]	[0.11]
**Health-related behavioural characteristics**								
Smoking status								
Non-smoker (ref)								
Current smoker	0.05	0.94**	-2.51***	-1.09***	-1.02***	-0.89***	0.03	-1.83***
	[0.20]	[0.31]	[0.35]	[0.23]	[0.17]	[0.17]	[0.20]	[0.17]
**Alcohol consumption**								
Non-drinker (ref)								
Current drinker	2.66***	3.43***	0.52	1.43***	-0.47***	0.49***	1.34***	0.33*
	[0.17]	[0.26]	[0.28]	[0.19]	[0.13]	[0.14]	[0.17]	[0.13]
**Physical activity**								
Less than the recommended level (ref)								
Recommended level	2.70***	3.80***	3.38***	3.34***	2.24***	3.85***	2.00***	3.61***
	[0.10]	[0.16]	[0.16]	[0.11]	[0.08]	[0.09]	[0.10]	[0.08]
**Work-related stressful life events**								
**Retiring from the workforce**								
No (ref)								
Yes	-0.63*	-1.77***	-1.87***	-0.50	0.06	0.35	-0.70**	-0.52**
	[0.25]	[0.46]	[0.46]	[0.28]	[0.18]	[0.20]	[0.25]	[0.19]
**Getting fired or made redundant**								
No (ref)								
Yes	0.59**	1.01**	-1.43***	-0.11	-0.79***	0.51**	0.59**	0.24
	[0.20]	[0.35]	[0.40]	[0.25]	[0.18]	[0.19]	[0.22]	[0.16]
**Major worsening in financial situation**								
No (ref)								
Yes	-0.95***	-4.12***	-8.15***	-5.81***	-5.59***	-3.86***	-1.11***	-2.03***
	[0.24]	[0.46]	[0.50]	[0.32]	[0.23]	[0.22]	[0.27]	[0.19]
**Family-related stressful life events**								
**Serious injury/illness to family member**								
No (ref)								
Yes	-0.09	-0.20	-1.77***	-1.14***	-0.98***	-0.86***	-0.21	-0.44***
	[0.09]	[0.19]	[0.19]	[0.12]	[0.08]	[0.09]	[0.11]	[0.08]
**Death of spouse or child**								
No (ref)								
Yes	0.05	-1.23	-6.72***	-4.94***	-5.09***	-2.65***	-0.43	0.01
	[0.45]	[0.77]	[0.87]	[0.54]	[0.37]	[0.36]	[0.43]	[0.31]
**Death of close relative/family member**								
No (ref)								
Yes	-0.13	-0.31	-1.32***	-1.10***	-0.79***	-0.46***	-0.28*	-0.25**
	[0.10]	[0.19]	[0.20]	[0.12]	[0.09]	[0.09]	[0.11]	[0.08]
**Personal stressful life events**								
**Personal injury or illness to self**								
No								
Yes	-6.21***	-18.88***	-8.11***	-11.03***	-3.23***	-5.12***	-10.34***	-5.69***
	[0.16]	[0.31]	[0.29]	[0.20]	[0.12]	[0.13]	[0.18]	[0.12]
**Experience of physical violence**								
No (ref)								
Yes	0.25	-2.16***	-6.54***	-4.48***	-3.78***	-1.81***	-1.65***	-1.14***
	[0.36]	[0.62]	[0.70]	[0.44]	[0.33]	[0.32]	[0.38]	[0.29]
Observations	230,462	230,462	230,462	230,462	230,462	230,462	230,462	230,462
Number of individuals	27,957	27,957	27,957	27,957	27,957	27,957	27,957	27,957
Other controls	Yes	Yes	Yes	Yes	Yes	Yes	Yes	Yes
Individual FE	Yes	Yes	Yes	Yes	Yes	Yes	Yes	Yes
Time FE	Yes	Yes	Yes	Yes	Yes	Yes	Yes	Yes

Note: (1) Robust standard errors in brackets. (2) ***, **, and * denote significance at the p < 0.01, *p < 0.05*,* andp < 0.1 levels*,* respectively.*

Table 5 presents the group comparison of the interaction effects between informal caregiving, disability status, and labour force participation on key HRQoL indicators: PCS, MCS, and SF-6D utility value. Individuals with both informal caregiving responsibilities and a disability exhibited significantly lower PCS scores, MCS scores, and SF-6D utility values compared to those without caregiving responsibilities and no disability. For example, individuals experiencing intensive caregiving responsibilities and had a disability demonstrated significantly lower scores on the PCS (β = -2.73, [0.023]), MCS (β = -2.96, [0.26]), and SF-6D utility value (β = -0.0391, [0.0026]) compared to their counterparts without caregiving responsibilities and no disability. The results also suggest that individuals facing the combined challenges of informal caregiving and unemployment/NLF experienced significantly lower HRQoL, as evidenced by lower scores on the MCS and SF-6D utility value, compared to those who did not provide care and were employed. For example, individuals with intensive caregiving responsibilities and unemployed/NLF status showed significantly lower MCS scores (β = -1.90, SE = 0.22) and SF-6D utility values (β = -0.0178, SE = 0.0023) compared to those without caregiving responsibilities and employed.

**Table 5:** Abridged regression results of group comparison in the interaction effects between informal caregiving, disability, and labour force status on the PCS, MCS, and SF-6D utility value

Model	(Model 1)	(Model 2)	(Model 3)	(Model 4)	(Model 5)	(Model 6)
Variables	PCS	PCS	MCS	MCS	SF-6D utility index	SF-6D utility index
**Group comparison in the interaction effects between informal caregiving and disability**						
Not a caregiver # No						
Not a caregiver # Yes	-3.23***		-1.53***		-0.0336***	
	[0.06]		[0.07]		[0.0007]	
Lighter caregiving # No	0.02		-0.40***		-0.0029**	
	[0.07]		[0.08]		[0.0009]	
Lighter caregiving # Yes	-2.79***		-1.57***		-0.0270***	
	[0.13]		[0.14]		[0.0016]	
Moderate caregiving # No	0.19		-0.75***		-0.0055***	
	[0.11]		[0.14]		[0.0015]	
Moderate caregiving # Yes	-3.00***		-1.76***		-0.0338***	
	[0.17]		[0.19]		[0.0019]	
Intensive caregiving # No	0.73***		-1.59***		-0.0086***	
	[0.20]		[0.25]		[0.0026]	
Intensive caregiving # Yes	-2.73***		-2.96***		-0.0391***	
	[0.23]		[0.26]		[0.0026]	
**Group comparison in the interaction effects between informal caregiving and labour force status**						
Not a caregiver # Employed (ref)						
Not a caregiver # Unemployed/NLF		-0.76***		-0.39***		-0.0104***
		[0.06]		[0.07]		[0.0008]
Lighter caregiving # Employed		0.08		-0.15		-0.0007
		[0.07]		[0.08]		[0.0009]
Lighter caregiving # Unemployed/NLF		-0.45***		-1.00***		-0.0087***
		[0.13]		[0.16]		[0.0017]
Moderate caregiving # Employed		0.12		-0.62***		-0.0056***
		[0.12]		[0.16]		[0.0017]
Moderate caregiving # Unemployed/NLF		-0.46**		-0.87***		-0.0114***
		[0.15]		[0.18]		[0.0017]
Intensive caregiving # Employed		0.61*		-1.61***		-0.0060
		[0.27]		[0.34]		[0.0033]
Intensive caregiving # Unemployed/NLF		-0.14		-1.90***		-0.0178***
		[0.20]		[0.22]		[0.0023]
Observations	230,462	230,462	230,462	230,462	230,462	230,462
Number of individuals	27,957	27,957	27,957	27,957	27,957	27,957
Other controls	Yes	Yes	Yes	Yes	Yes	Yes
Individual FE	Yes	Yes	Yes	Yes	Yes	Yes
Time FE	Yes	Yes	Yes	Yes	Yes	Yes

Notes: (1) Robust standard errors in brackets. (2) ***, **, and * denote significance at the p < 0.01, *p < 0.05*,* and p < 0.1 levels*,* respectively. (3) Abbreviations: ref*,* reference category; PCS*,* Physical Component Summary; MCS*,* Mental Component Summary; SF-6D*,* Short-Form Six-Dimension health index. (4) For a detailed list of control variables*,* please refer to* Table 3

Table 6 outlines the group comparisons of the interaction effects between informal caregiving, disability status, and labour force participation on the eight dimensions of the SF-36 health survey. Individuals with informal caregiving responsibilities and a disability exhibited significantly lower scores across all eight dimensions of the SF-36 health survey. For example, individuals who provided intensive care and experienced disability exhibited substantially lower scores on PF (β = -5.28), RP (β = -12.59), RE (β = -10.30), SF (β = -7.54), MH (β = -4.71), VT (β = -5.86), BP (β = -7.17), and GH (β = -5.90) compared to their counterparts with no caregiving responsibilities and without disability (Panel A). The results (Panel B) also showed that individuals providing intensive care while being unemployed or NLF exhibited significantly lower scores across all eight SF-36 domains: PF (β = -0.94), RP (β = -4.10), RE (β = -7.31), SF (β = -4.23), MH (β = -2.46), VT (β = -1.35), BP (β = -1.39), and GH (β = -0.71), compared to their employed counterparts with no caregiving responsibilities.

**Table 6:** Abridged regression results of group comparison in the interaction effects between informal caregiving, disability, and labour market status on the dimensions of the SF-36 scores

Panel A: Group comparison in the interaction effects between informal caregiving and disability								
Models	(Model 1)	(Model 2)	(Model 3)	(Model 4)	(Model 5)	(Model 6)	(Model 7)	(Model 8)
**Variables**	**PF**	**RP**	**RE**	**SF**	**MH**	**VT**	**BP**	**GH**
**Informal caregiving # Disability**								
Not a caregiver # No (ref)								
Not a caregiver # Yes	-5.83***	-11.67***	-6.58***	-5.75***	-2.73***	-4.31***	-7.05***	-5.81***
	[0.14]	[0.27]	[0.25]	[0.16]	[0.11]	[0.12]	[0.15]	[0.12]
Lighter caregiving # No	-0.07	-0.32	-0.90**	-0.77***	-0.70***	-0.28	-0.39*	-0.33*
	[0.17]	[0.29]	[0.29]	[0.19]	[0.13]	[0.15]	[0.18]	[0.13]
Lighter caregiving # Yes	-5.13***	-10.34***	-6.72***	-5.53***	-2.68***	-3.77***	-6.37***	-5.13***
	[0.31]	[0.57]	[0.57]	[0.35]	[0.23]	[0.25]	[0.32]	[0.24]
Moderate caregiving # No	0.22	-0.32	-1.89***	-1.49***	-1.02***	-0.52*	-0.41	-0.27
	[0.25]	[0.49]	[0.49]	[0.32]	[0.21]	[0.24]	[0.28]	[0.20]
Moderate caregiving # Yes	-5.32***	-11.90***	-7.28***	-6.11***	-3.05***	-3.96***	-6.64***	-5.68***
	[0.38]	[0.72]	[0.72]	[0.46]	[0.30]	[0.32]	[0.39]	[0.29]
Intensive caregiving # No	1.39**	0.21	-3.86***	-2.65***	-1.77***	-1.21**	-0.06	-0.56
	[0.45]	[0.90]	[0.92]	[0.58]	[0.38]	[0.43]	[0.49]	[0.37]
Intensive caregiving # Yes	-5.28***	-12.59***	-10.30***	-7.54***	-4.71***	-5.86***	-7.17***	-5.90***
	[0.53]	[0.93]	[0.98]	[0.62]	[0.41]	[0.42]	[0.52]	[0.39]
**Panel B: Group comparison in the interaction effects between informal caregiving and labour force status**								
Models	(Model 9)	(Model 10)	(Model 11)	(Model 12)	(Model 13)	(Model 14)	(Model 15)	(Model 16)
**Variables**	**PF**	**RP**	**RE**	**SF**	**MH**	**VT**	**BP**	**GH**
**Informal caregiving # Labour force status**								
Not a caregiver # Employed (ref)								
Not a caregiver # Unemployed/NLF	-1.85***	-4.01***	-3.56***	-2.22***	-0.53***	0.26*	-1.03***	-0.58***
	[0.15]	[0.25]	[0.26]	[0.17]	[0.12]	[0.13]	[0.15]	[0.12]
Lighter caregiving # Employed	0.05	0.21	-0.15	-0.30	-0.36**	0.06	-0.11	-0.04
	[0.17]	[0.30]	[0.30]	[0.19]	[0.13]	[0.15]	[0.18]	[0.13]
Lighter caregiving # Unemployed/NLF	-1.44***	-3.91***	-5.44***	-3.08***	-1.27***	0.04	-0.97**	-0.58*
	[0.33]	[0.57]	[0.60]	[0.38]	[0.25]	[0.26]	[0.33]	[0.24]
Moderate caregiving # Employed	-0.02	-0.27	-1.62**	-1.41***	-0.86***	-0.31	-0.30	-0.25
	[0.28]	[0.54]	[0.55]	[0.36]	[0.25]	[0.26]	[0.31]	[0.22]
Moderate caregiving # Unemployed/NLF	-1.11**	-4.35***	-4.80***	-2.87***	-1.16***	0.21	-0.88*	-0.56*
	[0.35]	[0.64]	[0.67]	[0.41]	[0.27]	[0.31]	[0.36]	[0.27]
Intensive caregiving # Employed	1.15	-1.39	-4.26***	-2.86***	-1.91***	-0.89	0.59	-0.91
	[0.59]	[1.16]	[1.22]	[0.77]	[0.52]	[0.55]	[0.61]	[0.50]
Intensive caregiving # Unemployed/NLF	-0.94*	-4.10***	-7.31***	-4.23***	-2.46***	-1.35***	-1.39**	-0.71*
	[0.47]	[0.81]	[0.87]	[0.54]	[0.35]	[0.38]	[0.46]	[0.35]
Observations	230,462	230,462	230,462	230,462	230,462	230,462	230,462	230,462
Number of individuals	27,957	27,957	27,957	27,957	27,957	27,957	27,957	27,957
Other controls	Yes	Yes	Yes	Yes	Yes	Yes	Yes	Yes
Individual FE	Yes	Yes	Yes	Yes	Yes	Yes	Yes	Yes
Time FE	Yes	Yes	Yes	Yes	Yes	Yes	Yes	Yes

Notes: (1) Robust standard errors in brackets. (2) ***, **, and * denote significance at the p < 0.01, *p < 0.05*,* and p < 0.1 levels*,* respectively. (3) Abbreviations: ref*,* reference category; NLF*,* Not in the Labour Force. (4) For a detailed list of control variables*,* please refer to* Table 3

### Robustness check

Table 7 presents the results of a sensitivity analysis that examines the effects of informal caregiving on HRQoL outcomes (PCS score, MCS score, SF-6D utility value, and eight dimensions of the SF-36 health survey) using fixed-effects models, excluding data from the COVID-19 pandemic period (waves 20 and 21). The sensitivity analysis results, which excluded data from the COVID-19 pandemic period (waves 20 and 21), further strengthen the robustness of our findings. The results were consistent with the primary analysis regarding effect size, direction, and statistical significance. Consistent with our primary regression analyses, the sensitivity analysis confirms that moderate and intensive caregiving significantly lower MCS scores, SF-6D utility values, and scores on key mental health-related SF-36 domains (RE, SF, MH, and VT).

**Table 7:** Estimated effects of informal caregiving on the PCS, MCS, and SF-6D utility value (without covid-period), abridged regression results

Models	(Model 1)	(Model 2)		(Model 3)	(Model 4)	(Model 5)	(Model 6)	(Model 7)	(Model 8)	(Model 9)	(Model 10)	(Model 11)
Variables	PCS	MCS	SF-6D		PF	RP	RE	SF	MH	VT	BP	GH
Informal caregiving												
Not a caregiver (ref)												
Lighter caregiving	0.24***	-0.22**	0.0011		0.42*	0.51	-0.35	-0.26	-0.32*	0.08	0.16	0.08
	[0.07]	[0.08]	[0.0009]		[0.17]	[0.29]	[0.30]	[0.19]	[0.13]	[0.14]	[0.18]	[0.13]
Moderate caregiving	0.30**	-0.52***	-0.0031*		0.52*	0.08	-1.16*	-1.02***	-0.72***	-0.04	0.04	-0.01
	[0.10]	[0.13]	[0.0013]		[0.24]	[0.45]	[0.46]	[0.29]	[0.20]	[0.22]	[0.25]	[0.18]
Intensive caregiving	0.70***	-1.58***	-0.0069**		1.14**	-0.04	-3.67***	-2.58***	-1.91***	-1.50***	-0.15	-0.16
	[0.17]	[0.21]	[0.0022]		[0.39]	[0.73]	[0.79]	[0.50]	[0.33]	[0.35]	[0.41]	[0.31]
Observations	199,462	199,462	199,462		199,462	199,462	199,462	199,462	199,462	199,462	199,462	199,462
Number of individuals	26,690	26,690	26,690		26,690	26,690	26,690	26,690	26,690	26,690	26,690	26,690
Other controls	Yes	Yes	Yes		Yes	Yes	Yes	Yes	Yes	Yes	Yes	Yes
Individual FE	Yes	Yes	Yes		Yes	Yes	Yes	Yes	Yes	Yes	Yes	Yes
Time FE	Yes	Yes	Yes		Yes	Yes	Yes	Yes	Yes	Yes	Yes	Yes

Notes: (1) Robust standard errors in brackets. (2) ***, **, and * denote significance at the p < 0.01, *p < 0.05*,* and p < 0.1 levels*,* respectively. (3) For a detailed list of control variables*,* please refer to* Table 3

### Heterogeneous effects

Sample heterogeneity can obscure genuine relationships and lead to erroneous conclusions. Therefore, it is essential to conduct subgroup analyses to explore potential differences within the sample and ensure an accurate interpretation of the results. Table 8 presents the effects of informal caregiving on PCS, MCS, and SF-6D utility values stratified by gender, allowing for the identification of potential gender-specific differences in these effects. The results showed that informal caregiving significantly reduced MCS scores for both men and women, although this reduction was higher for women. For instance, providing intensive caregiving lowers the MCS scores of male and female caregivers by 1.29 (β = − 1.29) and 1.64 units (β = − 1.64), respectively. The heterogeneity analysis highlights significant gender differences in the impact of informal caregiving on SF-6D utility values. Model 6 shows that moderate (β = -0.0043) and intensive caregiving (β = -0.0091) significantly reduce the utility value for female caregivers. However, informal caregiving did not considerably impact SF-6D utility values for male caregivers.

**Table 8:** Estimated effects of informal caregiving on the PCS, MCS, and SF-6D utility value by gender, abridged regression results

Models	(Model 1)	(Model 2)	(Model 3)	(Model 4)	(Model 5)	(Model 6)
Gender	Male	Female	Male	Female	Male	Female
Variables	PCS	PCS	MCS	MCS	SF-6D utility index	SF-6D utility index
Informal caregiving						
Not a caregiver (ref)						
Lighter caregiving	0.25**	0.07	-0.36***	-0.23*	0.0007	-0.0005
	[0.09]	[0.09]	[0.11]	[0.10]	[0.0013]	[0.0011]
Moderate caregiving	0.23	0.19	-0.39*	-0.64***	-0.0022	-0.0043**
	[0.15]	[0.13]	[0.18]	[0.16]	[0.0019]	[0.0015]
Intensive caregiving	0.61*	0.59**	-1.29***	-1.64***	-0.0041	-0.0091***
	[0.28]	[0.20]	[0.33]	[0.24]	[0.0036]	[0.0023]
Observations	107,638	122,824	107,638	122,824	107,638	122,824
Number of individuals	13,395	14,565	13,395	14,565	13,395	14,565
Other controls	Yes	Yes	Yes	Yes	Yes	Yes
Individual FE	Yes	Yes	Yes	Yes	Yes	Yes
Time FE	Yes	Yes	Yes	Yes	Yes	Yes

Note: (1) Robust standard errors in brackets. (2) ***, **, and * denote significance at the p < 0.01, *p < 0.05*,* and p < 0.1 levels*,* respectively. (3) For a detailed list of control variables*,* please refer to* Table 3

Table 9 shows the observed gender differences in the influence of informal caregiving on each of the eight dimensions of the SF-36 health survey. The heterogeneity analysis highlights the significant gender differences in the impact of informal caregiving on HRQoL, with female caregivers experiencing a more substantial negative impact. For example, intensive caregiving reduced RE (β = -4.18), SF (β = -1.96), and MH (β = -1.31) dimensions of the SF-36 health survey among males. Intensive caregiving, however, significantly reduces four SF-36 dimensions scores for females: RE (β = -3.64), SF (β = − 2.40), MH (β = − 2.22), and vitality (β = -1.79).

**Table 9:** Estimated effects of informal caregiving on the dimensions of the SF-36 by gender, abridged regression results

Panel A: Among male respondents only								
Models	(Model 1)	(Model 2)	(Model 3)	(Model 4)	(Model 5)	(Model 6)	(Model 7)	(Model 8)
Variables	PF	RP	RE	SF	MH	VT	BP	GH
**Informal caregiving**								
Not a caregiver (ref)								
Lighter caregiving	0.16	0.68	-0.65	-0.48	-0.65***	-0.01	0.08	-0.01
	[0.24]	[0.38]	[0.39]	[0.26]	[0.18]	[0.19]	[0.24]	[0.17]
Moderate caregiving	0.36	0.34	-1.23	-0.73	-0.56	0.15	-0.33	0.18
	[0.36]	[0.67]	[0.67]	[0.44]	[0.29]	[0.31]	[0.38]	[0.26]
Intensive caregiving	0.88	0.47	-4.18**	-1.96*	-1.31*	-0.73	-0.09	-0.30
	[0.66]	[1.18]	[1.31]	[0.83]	[0.53]	[0.56]	[0.69]	[0.52]
Observations	107,638	107,638	107,638	107,638	107,638	107,638	107,638	107,638
Number of individuals	13,395	13,395	13,395	13,395	13,395	13,395	13,395	13,395
Other controls	Yes	Yes	Yes	Yes	Yes	Yes	Yes	Yes
Individual FE	Yes	Yes	Yes	Yes	Yes	Yes	Yes	Yes
Time FE	Yes	Yes	Yes	Yes	Yes	Yes	Yes	Yes
**Panel B: Among female respondents only**								
**Models**	**(Model 1)**	**(Model 2)**	**(Model 3)**	**(Model 4)**	**(Model 5)**	**(Model 6)**	**(Model 7)**	**(Model 8)**
**Variables**	**PF**	**RP**	**RE**	**SF**	**MH**	**VT**	**BP**	**GH**
**Informal caregiving**								
Not a caregiver (ref)								
Lighter caregiving	0.17	-0.22	-0.67	-0.45	-0.32*	-0.05	-0.16	-0.03
	[0.20]	[0.38]	[0.39]	[0.24]	[0.16]	[0.18]	[0.22]	[0.16]
Moderate caregiving	0.33	-0.68	-1.54**	-1.26***	-0.85***	-0.39	0.05	-0.33
	[0.29]	[0.54]	[0.56]	[0.35]	[0.24]	[0.26]	[0.30]	[0.23]
Intensive caregiving	0.96*	-0.93	-3.64***	-2.40***	-2.22***	-1.79***	-0.17	-0.41
	[0.45]	[0.84]	[0.88]	[0.55]	[0.36]	[0.39]	[0.46]	[0.35]
Observations	122,824	122,824	122,824	122,824	122,824	122,824	122,824	122,824
Number of individuals	14,565	14,565	14,565	14,565	14,565	14,565	14,565	14,565
Other controls	Yes	Yes	Yes	Yes	Yes	Yes	Yes	Yes
Individual FE	Yes	Yes	Yes	Yes	Yes	Yes	Yes	Yes
Time FE	Yes	Yes	Yes	Yes	Yes	Yes	Yes	Yes

Notes: (1) Robust standard errors in brackets. (2) ***, **, and * denote significance at the p < 0.01,*p  < 0.05*,* and p < 0.1 levels*,* respectively. (3) For a detailed list of control variables*,* please refer to* Table 3

## Discussion

### Key findings

The primary objective of our study was to examine the effects of informal caregiving on multiple aspects of HRQoL, including the PCS, MCS, SF-6D utility index, and the eight individual dimensions of the SF-36 health survey. Longitudinal data from the most recent 16 waves of the HILDA Survey, spanning from 2006 to 2021, were analysed. Given the longitudinal nature of the data, fixed-effects regression models were utilised to examine the effects of informal caregiving on HRQoL. Our findings suggest that informal caregiving has a detrimental impact on HRQoL. Our findings showed that regardless of caregiving intensity [lighter (< 5 h/week), moderate (5–19 h/week), or intensive (≥ 20 h/week)], caregivers experienced a significant reduction in MCS scores. Our results also indicated that both moderate (5–19 h/week) and intensive (≥ 20 h/week) levels of caregiving had a significant negative impact on SF-6D utility values. Extending our analysis to the individual SF-36 dimensions, we found that intensive caregiving (≥ 20 h/week) had a significant negative impact on Role Emotional Functioning (RE), Social Functioning (SF), Mental Health (MH), and Vitality (VT). Similar to intensive caregiving, lighter (< 5 h/week) and moderate (5–19 h/week) levels of caregiving also had a significant negative impact on three subscales of the SF-36 related to mental health (RE, SF, and MH).

In summary, our results demonstrate that increased caregiving burden is associated with significant deterioration across multiple dimensions of HRQoL as measured by the SF-36. Furthermore, the magnitude of the negative impact on HRQoL increased with higher levels of caregiving. Caregivers providing intensive care (≥ 20 h/week) and moderate care (5–19 h/week) experienced greater reductions in various HRQoL measures compared to those providing lighter care (< 5 h/week). These findings are in line with previous research in this area. Previous studies investigating the impact of informal caregiving on HRQoL have generally demonstrated similar negative correlations [15, 19, 31–37]. Studies from diverse global regions, including Europe, Asia, and Africa, have consistently reported lower HRQoL among informal caregivers [31, 32, 34, 35]. Previous Australian studies utilising earlier waves of the HILDA Survey have also reported negative impacts of informal caregiving on mental health, general health, and vitality [15, 36, 37]. Past studies have also reported lower levels of self-rated well-being among informal caregivers [19]. However, findings regarding the impact of informal caregiving on physical functioning have been mixed. A recent study from Australia reported no impact of informal caregiving on PF. However, an earlier study noted improvements in PF scores for non-employed male caregivers after two years of initiation of caregiving responsibilities [36, 37]. An earlier study in Germany reported no significant impact of informal caregiving on physical and mental health while observing a decline in life satisfaction [9]. We observed a positive relationship between caregiving and physical functioning. While some studies have reported mixed findings, the overall body of research within and outside Australia suggests a consistent negative association between the intensity of informal caregiving and HRQoL [31–35, 37, 49]. Our findings further support this conclusion, demonstrating that higher levels of caregiving are associated with more significant declines in HRQoL. Our findings provide valuable insights into gender-specific differences in the impact of informal caregiving on HRQoL. Heterogeneous analyses revealed substantial gender differences, with females experiencing more pronounced declines in HRQoL across multiple domains compared to males in response to informal caregiving responsibilities. For example, our analysis revealed a significant negative association between moderate and intensive levels of caregiving and SF-6D utility values among women. However, no significant association was found between caregiving intensity and SF-6D utility values among men. The observed gender differences in the impact of caregiving on HRQoL are highly consistent with findings from previous research, which has consistently shown that women are disproportionately affected by the negative health consequences of caregiving [20–26, 32, 36, 49]. Previous research offers some plausible explanations for the findings observed in our study. Prior research has demonstrated that stress, emotional strain, and depressive symptoms arising from caregiving responsibilities can significantly impact mental health and lower health state utility value [8, 10–12, 19]. Informal caregiving often necessitates a significant compromise in personal time, leading to reduced engagement in leisure activities and social interactions, which can adversely impact social and emotional well-being [6, 17, 22, 27, 50, 51]. There is also evidence that the presence of an ill family member in need of assistance can increase the prevalence of depressive symptoms among other household members [52, 53]. Caregiving tasks may not always be physically demanding, but they can impose significant emotional and mental stress on caregivers [53]. This aligns with the observation that informal caregiving primarily impacts mental health rather than physical health.

### Policy implications

The findings of our study have significant implications for the development and implementation of effective public health policies aimed at supporting the well-being of informal caregivers. It is crucial to recognise that, in addition to the well-documented economic costs, informal caregiving carries substantial non-economic costs that cannot be overlooked. Our study highlights the disutility and negative mental health impacts associated with informal caregiving, particularly among female caregivers. With the increased need for informal caring, it is crucial to acknowledge the hidden costs borne by informal caregivers and develop preventative strategies. Our findings underscore the need for policymakers to prioritise the development and implementation of support services aimed at alleviating the burden of caregiving and mitigating the associated anxiety, stress, and decreased emotional well-being. Some policy interventions that might be helpful or protective for the social and emotional well-being of informal carers include providing more financial support and security, increasing access to community services (e.g., peer support groups), ensuring more flexible working patterns for caregivers, increasing access to information and training and growing respite care opportunities.

Our findings are crucial for health technology assessment (HTA) practitioners conducting economic evaluations of interventions to improve caregiver well-being. Economic evaluation involves comparing the costs and benefits of proposed interventions and guides efficient resource allocation. Our findings demonstrate the disutility associated with different levels of informal caregiving. The findings regarding the disutility associated with informal caregiving can be valuable for conducting cost-effectiveness analyses of interventions aimed at improving caregiver well-being.

### Strengths, limitations, and avenues for further research

Our study has several notable strengths. Firstly, our empirical analysis utilised data from a nationally representative longitudinal survey, enabling us to follow individuals over time and generate precise estimates. Secondly, by utilizing fixed-effects regression models, we could account for unobserved individual-level heterogeneity, leading to more robust and reliable estimates than traditional cross-sectional analyses. Thirdly, using the well-established and widely validated SF-36 health survey to measure HRQoL, we ensured high reliability and validity in assessing the association between informal caregiving and HRQoL. Fourthly, our study findings, including estimates of the disutility associated with different levels of informal caregiving, are critical for informing the cost-effectiveness analyses of interventions to improve caregiver well-being.

Despite the strengths mentioned above, our study has two significant limitations that must be acknowledged. First, it is essential to note that using an unbalanced panel dataset presents limitations in establishing causal inferences. Therefore, the estimates presented in our study should be interpreted as associations between informal caregiving and HRQoL rather than definitive causal effects. Second, reliance on self-reported data for key variables, including HRQoL and caregiving activities, may introduce potential biases, such as recall bias and social desirability bias, which could potentially influence our study findings. A further limitation of our study is its sole reliance on the generic SF-36 Health Survey to assess HRQoL without incorporating carer-specific quality of life (QoL) measures like the CES, CarerQoL, or ASCOT-Carer. These measures capture different constructs, but they are all designed to be broader than HRQoL to ensure consideration of the impacts of caring or carer support beyond health. These specialised instruments could have offered a more nuanced understanding of the caregiver experience, particularly regarding stress, financial strain, and emotional well-being.

Despite the limitations inherent in our study, our findings provide valuable insights and suggest several promising avenues for future research in this area. Future research should delve deeper into the complexities of informal caregiving by investigating the impact of various factors, including caregiver personality traits (e.g., agreeableness), the specific type of caregiving provided (e.g., care for elderly, children, or palliative patients), and the unique dynamics of the caregiver-recipient relationship, on the HRQoL outcomes of caregivers. This approach may help identify specific groups of caregivers who may be experiencing greater challenges and require more targeted support services. Future research should also investigate the mechanisms through which informal caregiving impacts HRQoL in different caregiver groups.

## Conclusion

Our study examines the effects of varying levels of informal caregiving on HRQoL. Our findings highlight a significant reduction in the SF-6D utility value of individuals providing informal care. Our findings also demonstrate that both moderate and intensive levels of caregiving had a significant negative impact on the mental health-related dimensions of the SF-36 and the MCS score. Additionally, our study revealed significant gender differences in the impact of informal caregiving on HRQoL, with female caregivers experiencing more pronounced declines in HRQoL than male caregivers.

Our research extends existing knowledge by demonstrating the direct negative impact of informal caregiving on caregivers’ HRQoL. Our findings, particularly quantifying the disutility associated with different levels of informal caregiving, have significant implications for the economic evaluation of interventions designed to support informal carers and improve their health outcomes. Further research is crucial to fully understand the complex mechanisms through which informal caregiving impacts different domains of HRQoL. A better understanding of these mechanisms is essential for developing more effective policy interventions to support the health and well-being of informal caregivers.

## Electronic supplementary material

Below is the link to the electronic supplementary material.

Supplementary Material 1

## Data Availability

There are two versions of the HILDA data: the General Release and the Restricted Release. This study utilised restricted release data from the Household, Income and Labour Dynamics in Australia (HILDA) Survey. Funded by the Australian Government Department of Social Services (DSS), the Survey is managed by the Melbourne Institute of Applied Economic and Social Research (MI) at the University of Melbourne. Access to the complete HILDA dataset is limited and requires specific approval, as it contains sensitive personal information. To apply for access to any of the DSS Longitudinal Studies datasets, first, all applicants and collaborators who need to view unit record data must complete and sign a once only Confidentiality Deed Poll and email the scanned, signed copy to DSS (DataAccess@dss.gov.au) and ADA (ada@ada.edu.au). Electronic signatures are currently accepted. Detailed information regarding data access procedures and requirements can be found at https://dataverse.ada.edu.au/dataverse.xhtml?alias=hilda.

## Abbreviations

HILDA: Household, Income and Labour Dynamics in Australia Survey
HRQoL: Health-related Quality of Life
MCS: Mental Component Summary
PCS: Physical Component Summary
SF-6D: Short Form-6D Utility Index
SF-36: The Short Form-36 Health Survey
