# Supplementary material for: Deterioration of health-related quality of life: the hidden health burden of informal caregiving
Source: Eur J Health Econ. 2025 Apr 17;26(8):1415–38. doi: 10.1007/s10198-025-01776-5 (PMC12572017; doi:10.1007/s10198-025-01776-5)
Supplement: Supplementary file 1 — Supplementary Material 1 [file 10198_2025_1776_MOESM1_ESM.docx]

**Appendix**


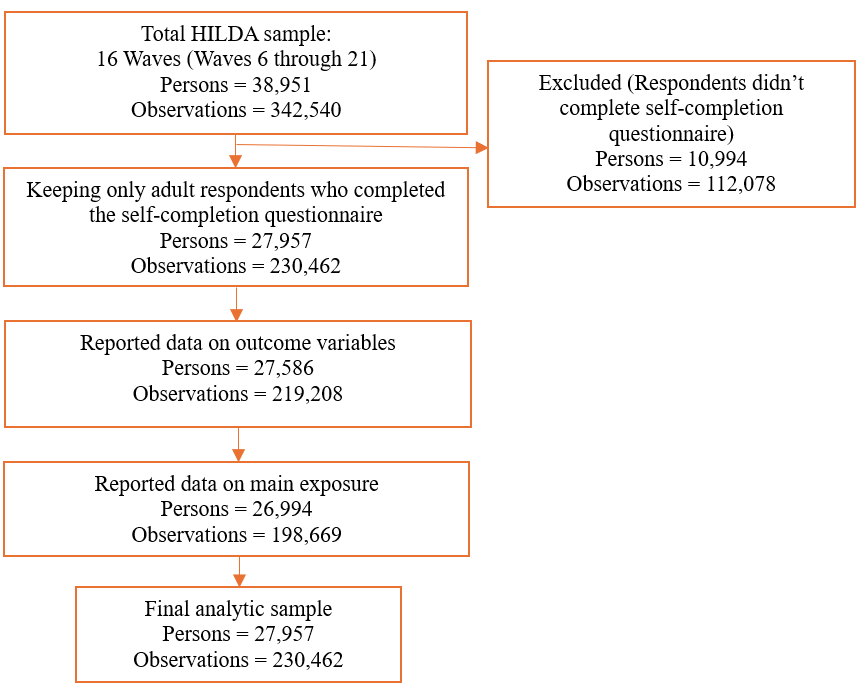


Notes: 1. We did not drop observations due to missingness in outcome, key exposure, and control variables. To ensure the replicability of our analysis and minimise potential bias due to missing data, we employed imputation techniques to replace missing values.

Figure A1: Flow chart showing the selection of HILDA participants for the analyses.

Table A1: Missing observation analysis

| **Variables** | **Missing Observations** | **Total observations** | **Percent (%) Missing** |
| --- | --- | --- | --- |
| Physical functioning | 2,853 | 230,462 | 1.24 |
| Role physical | 3,066 | 230,462 | 1.33 |
| Role emotional | 3,273 | 230,462 | 1.42 |
| Social functioning | 276 | 230,462 | 0.12 |
| Mental health | 1,346 | 230,462 | 0.58 |
| Vitality | 1,321 | 230,462 | 0.57 |
| Bodily pain | 1,666 | 230,462 | 0.72 |
| General health | 2,864 | 230,462 | 1.24 |
| Sf-6D utility value | 9,871 | 230,462 | 4.28 |
| Informal caregiving | 22,986 | 230,462 | 9.97 |
| Age | 0 | 230,462 | 0 |
| Gender | 0 | 230,462 | 0 |
| Relationship status | 12 | 230,462 | 0.01 |
| Highest education level completed | 0 | 230,462 | 0 |
| Labour market status | 0 | 230,462 | 0 |
| Household yearly disposable income | 0 | 230,462 | 0 |
| Indigenous status | 101 | 230,462 | 0.04 |
| Region of residence | 35 | 230,462 | 0.02 |
| Weight category (BMI) | 10,750 | 230,462 | 4.66 |
| Disability | 57 | 230,462 | 0.02 |
| Smoking status | 2,360 | 230,462 | 1.02 |
| Alcohol consumption | 2,440 | 230,462 | 1.06 |
| Physical Activity | 1,374 | 230,462 | 0.6 |
| Retiring from the workforce | 2,628 | 230,462 | 1.14 |
| Getting fired or made redundant | 2,782 | 230,462 | 1.21 |
| Major worsening in financial situation | 2,643 | 230,462 | 1.15 |
| Serious injury or illness to a family member | 2,998 | 230,462 | 1.3 |
| Death of spouse or child | 2,808 | 230,462 | 1.22 |
| Death of a close relative/family member | 2,677 | 230,462 | 1.16 |
| Experience of physical violence | 2,823 | 230,462 | 1.22 |
| Personal injury or illness to self | 2,893 | 230,462 | 1.26 |

Table A2: Description of the control variables

| **Variables** | **Measure** |
| --- | --- |
| **Socio-demographic characteristics** | |
| Age | 0 = 15-24 (Youth), 1 = 25-39 (Young adult), 2 = 40-64 (Middle-aged adult), 3 = ≥ 65 years (Older adult). |
| Gender | 0 = Male, 1 = Female. |
| Relationship status | 0 = Partnered (married in a registered marriage, and never married but living with someone in a relationship), 1 = Unpartnered (never married and not living with someone in a relationship, separated but not divorced, divorced, and widowed). |
| Highest education  level completed | 0 = Year 12 and below (year 12, and year 11 and below), 1 = Professional qualifications (advanced diploma or diploma, and certificate III or IV), 2 = University qualifications (postgraduate – masters or doctorate, graduate diploma or certificate, bachelor or honours). |
| Household yearly  disposable income | 0 = Quintile 1 (poorest), 1 = Quintile 2 (poorer), 2 = Quintile 3 (middle), 3 = Quintile 4 (richer), 4 = Quintile 5 (richest). |
| Labour market status | 0 = Employed, 1 = Unemployed/Not in the labour force (NLF). |
| Indigenous status | 0 = Not of Indigenous origin, 1 = Indigenous origin (Aboriginal, Torres Strait Islander, and both Aboriginal and Torres Strait Islander). |
| Region of residence | 0 = Major city, and 1 = Regional or remote area (inner regional, outer regional, remote, and very remote Australia). |
| **Health-related characteristics** | |
| Weight category | 0 = Underweight (BMI<18.50), 1 = Healthy weight (BMI 18.50-24.99), 2 = Overweight or pre-obese (BMI 25.00-29.99), 3 = Obesity (BMI ≥ 30). |
| Long-term health condition or disability | 0 = No, 1 = Yes. |
| **Health-related behaviours** | |
| Smoking status | 0 = Non-smoker (Never smoked, and former smoker), 1 = Current smoker (smokes daily, smokes at least weekly, and smokes less often than weekly). |
| Alcohol consumption | 0 = Non-drinker (Never drunk, and Ex-drinker), 1 = Current drinker (only rarely, 1-2 days, 2-3 days, 3-4 days, 5-6 days per week and every day). |
| Physical activity | 0 = Less than the recommended level (not at all, less than once, 1 to 2, and 3 times a week), 1= Recommended level (> 3 times a week and every day). |
| **Work-related stressful life events** | |
| Retiring from the workforce | 0 = No, 1 = Yes. |
| Getting fired or made redundant | 0 = No, 1 = Yes. |
| Major worsening in financial situation | 0 = No, 1 = Yes. |
| **Family-related stressful life events** | |
| Serious injury/illness to family member | 0 = No, 1 = Yes. |
| Death of spouse or child | 0 = No, 1 = Yes. |
| Death of a close relative/family member | 0 = No, 1 = Yes. |
| **Personal stressful life events** | |
| Experience of physical violence | 0 = No, 1 = Yes. |
| Personal injury or illness to self | 0 = No, 1 = Yes. |

Notes: 1. The study used a ‘modified OECD’ equivalence scale to measure equivalised household annual disposable income.

Table A3: Estimated transition rate for each level of informal caregiving (from T to T+ 1+...+n).

| **Informal caregiving** | **Informal caregiving** | | | | **Total** |
| --- | --- | --- | --- | --- | --- |
|  | Not a caregiver,  n (%) | Lighter caregiving,  n (%) | Moderate caregiving,  n (%) | Intensive caregiving,  n (%) |  |
| Not a caregiver | 167,093 (93.92) | 7,571 (4.26) | 2,450 (1.38) | 787 (0.44) | 177,901 (100) |
| Lighter caregiving | 6,133 (44.51) | 6,424 (46.62) | 1,073 (7.79) | 150 (1.09) | 13,780 (100) |
| Moderate caregiving | 2,324 (32.61) | 982 (13.78) | 3,067 (43.03) | 754 (10.58) | 7,127 (100) |
| Intensive caregiving | 828 (22.40) | 150 (4.06) | 621 (16.80) | 2,098 (56.75) | 3,697 (100) |
| **Total** | 176,378 (87.10) | 15,127 (7.47) | 7,211 (3.56) | 3,789 (1.87) | 202,505 (100) |

Notes:1. T indicates the timepoint. 2. The total number of yearly observations used to calculate the transition rate is 202,505.
